# Ephemeral Speciation in a New Guinean Honeyeater Complex (*Aves*: *Melidectes*)

**DOI:** 10.1111/mec.17760

**Published:** 2025-04-11

**Authors:** Ingo A. Müller, Filip Thörn, Samyuktha Rajan, Remi‐André Olsen, Per G. P. Ericson, Valentina Peona, Brian Tilston Smith, Gibson Maiah, Bonny Koane, Bulisa Iova, Mozes P. K. Blom, Martin Irestedt, Knud A. Jønsson

**Affiliations:** ^1^ Department of Bioinformatics and Genetics Swedish Museum of Natural History Stockholm Sweden; ^2^ Department of Zoology Stockholm University Stockholm Sweden; ^3^ Museum für Naturkunde, Leibniz Institut für Evolutions‐ Und Biodiversitätsforschung Berlin Germany; ^4^ Science for Life Laboratory, Department of Biochemistry and Biophysics Stockholm University Solna Sweden; ^5^ Swiss Ornithological Institute Vogelwarte Sempach Switzerland; ^6^ Department of Ornithology American Museum of Natural History New York New York USA; ^7^ New Guinea Binatang Research Centre Madang Papua New Guinea; ^8^ Papua New Guinea National Museum and Art Gallery Port Moresby Papua New Guinea

**Keywords:** birds, hybridisation, molecular evolution, phylogeography, population genetics—empirical, speciation

## Abstract

Speciation is a fundamental concept in evolutionary biology, and understanding the mechanisms driving speciation remains the foremost research topic within this field. Hybridisation is often involved in speciation and can influence its rates, potentially accelerating, decelerating or even reversing the process. This study investigates the evolutionary history of the New Guinean bird genus *Melidectes*, consisting of six species that inhabit various montane regions at different elevations. While most *Melidectes* species have allopatric distributions, two species overlap in the central mountain range and hybridise. However, plumage differences and elevational adaptations are assumed to maintain the species' boundaries. Utilising specimens from natural history collections and comprehensive genomic analyses, including a de novo genome assembly, we characterise allopatric speciation patterns within the genus and highlight how future speciation could potentially be driven by climate change. Contrary to previous hypotheses, our findings suggest that in the two distributionally overlapping species, phenotypic differences do not prevent gene flow. We find limited acoustic differentiation and extensive admixture across most of their distributions. Divergence and admixture levels conform poorly to the current taxonomy and follow a geographical pattern in which the most isolated populations at the ends of the distributions are most divergent and show least admixture. However, in contrast, their mitochondrial genomes do group in accordance with species identity, namely, into two deeply divergent lineages. We propose that this system demonstrates the ephemeral nature of speciation, in which two incipient species have started mixing extensively as they came into secondary contact, resulting in nearly complete fusion into a single lineage.

## 1 Introduction

A long‐standing question in evolutionary biology is to understand the underlying mechanisms that lead to speciation (Coyne 1992; Turelli et al. 2001). To this end, analyses of the interactions of closely related species and populations are important to determine how species barriers are formed and maintained in space and through time. Hybridising taxa, where individuals from genetically distinct populations mate with each other (Allendorf et al. 2001), offer a great opportunity to analyse speciation in progress (Barton and Hewitt 1989). Traits and genes involved in the speciation process can be identified more easily in hybridising systems compared to systems in which species are allopatrically distributed or completely reproductively isolated, since these systems may exhibit distinct patterns of differentiation especially at barrier loci (Ravinet et al. 2017). At the same time, one may expect that extensive hybridisation between species should lead to homogenisation of the genomes and breakdown of the species barrier (Coyne 2007; Rosenblum et al. 2012). However, across many organismal groups, examples exist of hybridising taxa for which species remain genetically distinct despite high levels of gene flow, e.g., Hagberg et al. (2022), Kraus et al. (2012), Morgan et al. (2012) or Poelstra et al. (2014).

There is an increased understanding that hybridisation is often an important part of speciation (Abbott et al. 2013) and can even accelerate it in some cases—for example, through non‐viability or sterility of hybrids, which may strengthen pre‐mating reproductive barriers (Presgraves et al. 2003). Therefore, the majority of studies consider hybridisation a transient stage towards full speciation, despite several examples showing that hybridisation can also reverse speciation (Garrick et al. 2014; Kleindorfer et al. 2014; Seehausen et al. 2008; Taylor et al. 2006). Under the ephemeral speciation model (Rosenblum et al. 2012), incipient speciation is hypothesised to be a very common process, but incipient lineages almost never persist long enough to become distinct species. By considering lineage fusions as a potential evolutionary force, one may gain a more complete picture of speciation histories, as has been done, for example, in ravens (Kearns et al. 2018; Webb et al. 2011). The challenge lies in obtaining evidence of past ephemeral speciation, as completed fusions may have entirely homogenised the genomes and thus removed any signal of past differentiation. One way that past ephemeral speciation may still be detectable is from genomic regions that underwent independent evolutionary histories, such as mitochondrial DNA, as exhibited in ravens (Kearns et al. 2018).

Birds represent an ideal study system to address questions related to the role of hybridisation in speciation, including potential lineage fusions, as extensive hybridisation (> 1700 bird species or approximately 16% of all described avian species) between species is known from across the avian tree of life (Ottenburghs et al. 2015). Within birds, New Guinean honeyeaters of the genus *Melidectes* offer an interesting system, as intricate elevational hybridisation patterns have been suggested through morphological assessments (Gilliard 1959; Mayr and Gilliard 1952). *Melidectes* includes six species that inhabit montane regions across New Guinea ( *M. torquatus* [Ornate Melidectes], *M. leucostephes* [Vogelkop Melidectes], *M. ochromelas* [Cinnamon‐browed Melidectes], *M. foersteri* [Huon Melidectes], *M. rufocrissalis* [Yellow‐browed Melidectes], *M. belfordi* [Belford's Melidectes]). Some species have broad distributions, whereas others are confined to narrow elevational bands and small isolated mountain ranges. In some montane regions, several members of *Melidectes* co‐occur, but they are often elevationally separated (Del Hoyo et al. 2008; Diamond 1973). These taxa were described to often exhibit narrow hybrid zones (Mayr and Gilliard 1952) with the exception of *M. belfordi* and *M. rufocrissalis*, which tend to have a broad zone of elevational overlap. The two species are known to hybridise, and morphological intermediates are regularly observed in the wild and in museum collections (Mayr and Gilliard 1952, 1954). *Melidectes belfordi* is distributed throughout most of the central mountain range of New Guinea, while *M. rufocrissalis* only occurs in eastern New Guinea. A few other species pairs of *Melidectes* also have distributional overlaps, but no hybrids have been documented. The phylogenetic relationships between *M. foersteri* , *M. belfordi* and *M. rufocrissalis* are currently unclear, possibly due to hybridisation that occurs within this group (Marki et al. 2017).

In this study, we use a museomic approach to generate a large dataset of whole genomes to analyse past and potential future speciation processes and the role of hybridisation within *Melidectes*. We expected to observe evidence of allopatric speciation in isolated regions of New Guinea such as the Huon or Bird's Head peninsulas but also between taxa that are segregating across elevational gradients, both of which have previously been described in other avian groups (Pujolar et al. 2022; Stelbrink et al. 2022). We further hypothesised that levels of hybridisation are correlated with geographic and elevational overlap. Furthermore, we tested whether isolated taxa exhibit stronger genetic differentiation than taxa with overlapping distributions.

## 2 Materials and Methods

### 2.1 De Novo Reference Assembly of *Melidectes torquatus*

We assembled a new genome for *Melidectes torquatus* to serve as our reference genome for population genetic analyses as it has been previously identified as an outgroup for the whole genus (Marki et al. 2017). DNA was extracted from a blood sample of specimen NHMD 616058 by applying the Thermo Scientific KingFisher Cell and Tissue DNA Kit on a KingFisher Duo Prime instrument. Sequencing libraries were prepared and sequenced at the National Genomics Infrastructure (NGI) in Stockholm resulting in three sets of data that were used to assemble the genome. (1) Long‐read sequences were obtained from Oxford Nanopore Technologies' (ONT) PromethION run on three R9 flow cells, from three gDNA preps using the SQK‐LSK110 protocol, (2) Omni‐C data was generated from a Dovetail Omni‐C library prepared from frozen and ground muscle tissue, sequenced on an Illumina NovaSeq 6000 platform and (3) paired 2 × 100 bp reads using Illumina NovaSeq 6000 machines on S4 flow cells. Omni‐C and paired Illumina reads were used to bridge contigs and scaffolds obtained from ONT's long reads to create a more contiguous assembly.

Using the ONT data, we used *Guppy* (v5.0.12) for basecalling and applied the super high accuracy (SUP) model which gives the lowest error rates of the models supplied with the software. The first draft assembly was generated using *flye* (v2.9.1) with the parameter ‘—no‐alt‐contigs’ (Kolmogorov et al. 2019), and was subsequently polished with *hypo* (v1.0.3, (Kundu et al. 2019)). Scaffolding and the final curated assembly were obtained through *YaHS* (see Table S1 for specific github tree, (Zhou et al. 2022)), *pairtools* (v0.3.0, (Open2C et al. 2024)) and *Juicebox* (v1.11.08, (Dudchenko et al. 2018)). The assembly quality was evaluated at different stages using *busco* (v5.3.1, (Manni et al. 2021)), *merqury* (v1.3, (Rhie et al. 2020)) and *quast* (v5.0.2, (Mikheenko et al. 2018)). Resulting scaffolds of the final assembly were linked to chromosomes by aligning them against chromosome‐level assemblies of the Helmeted Honeyeater ( *Lichenostomus melanops cassidix* , ENA: GCA_008360975.1) and Zebra Finch ( *Taeniopygia guttata* , ENA: GCA_003957565.4) using *minimap2* (v2.24, (Li 2018)). Based on these alignments, we identified 41 distinct scaffolds from this assembly and designated this as the reference genome. Additionally, we generated a de novo repeat library on top of the reference genome using *RepeatModeler2* (v2.0.5, (Flynn et al. 2020)) and then used it to mask the genome with *RepeatMasker* (v4.1.5, (Smit et al. 2013)).

### 2.2 DNA Sampling, Sequencing and Read Processing

**FIGURE 1:**
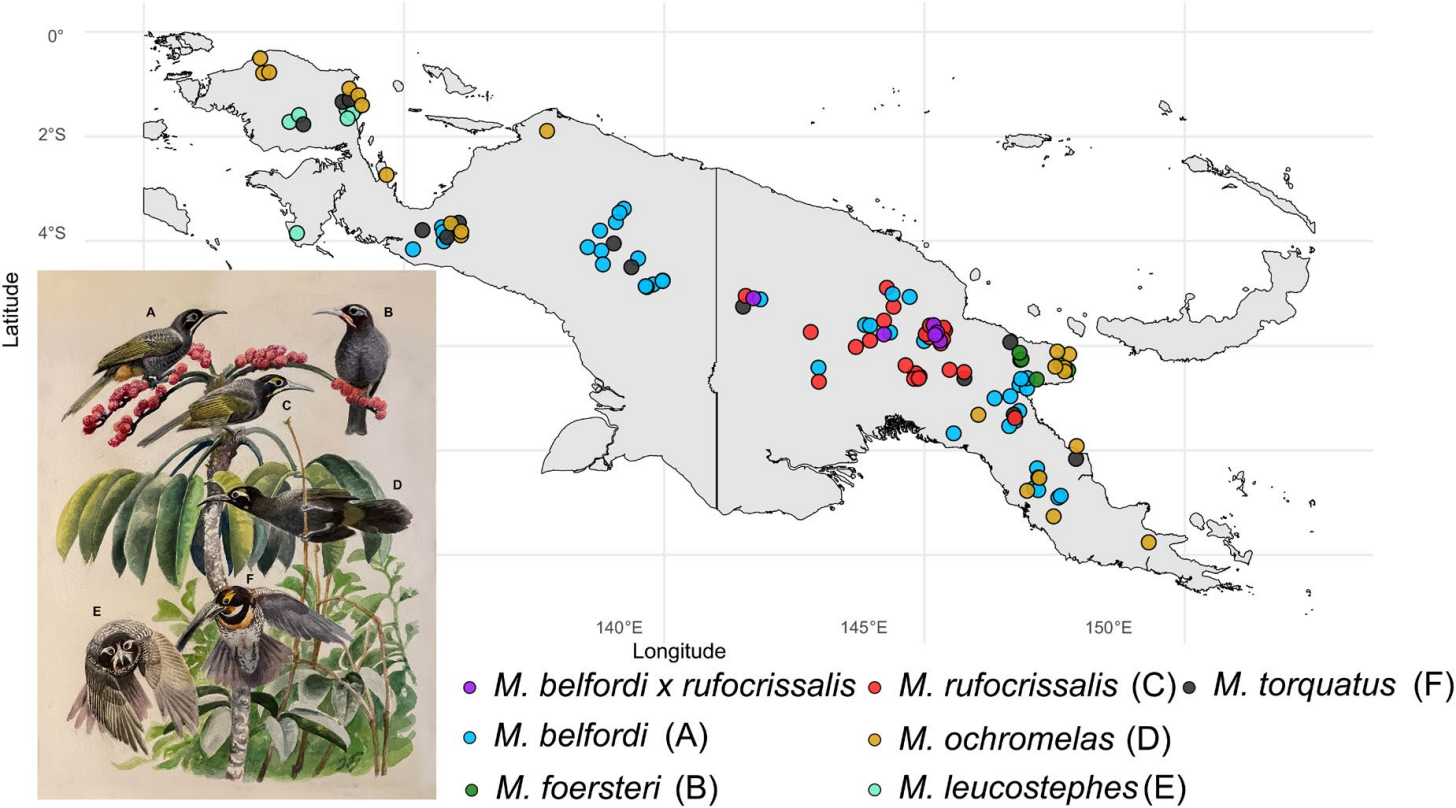
Distribution of sampling sites. Individuals are slightly jittered to differentiate samples from the same locality. Morphological hybrids between *M. belfordi* and *M. rufocrissalis* are shown in purple. Bottom left: Illustrations of all six *Melidectes* species by Jon Fjeldså.

Taxonomic classifications in this study follow the IOC World Bird List (Gill et al. 2023). Samples used for resequencing were collected from 124 individuals across all six species of *Melidectes* ( *M. torquatus* , *M. leucostephes* , *M. ochromelas* , *M. foersteri* , *M. rufocrissalis* , *M. belfordi*, as well as 6 individuals that were identified as morphological hybrids between *M. rufocrissalis* and *M. belfordi* and 5 more presumed hybrid individuals), comprising 106 toepads from historical specimens and 18 fresh tissue extracts. See Table S4 for a complete list of all included individuals and Figure 1 for sampling sites. Figures S1–S6 show the distribution of each species and highlight important regions discussed in this study.

For fresh samples, Qiagen DNeasy Blood and Tissue kits were applied for DNA extraction. We extracted DNA and prepared sequencing libraries from historical samples according to a protocol adapted from Meyer and Kircher (2010) which has been shown to be effective for avian museum specimens as seen in Irestedt et al. (2022). DNA was isolated from toepads by adhering to Qiagen's guidelines for animal tissues but also introducing dithiothreitol (DTT) to enhance the ligation yield. USER enzyme was added during the library preparation phase to minimise the deamination patterns that are often found in ancient or historical fragmented DNA (Briggs et al. 2010). For samples from museum collections, we prepared four libraries for each sample to improve library complexity, while a single library was prepared for fresh tissue samples. Each library contained a unique pair of dual indexes which allowed for pooling of the samples. A comprehensive overview on the methodology can be found in Irestedt et al. (2022). Whole genome resequencing was run on Illumina NovaSeq 6000 platforms with S4 flow cells for 200 cycles, producing reads of 2 × 100 bp. This was carried out at the National Genomics Infrastructure in Stockholm, with up to 96 libraries from 24 samples sharing a single flow cell lane.

Post‐sequencing, we polished reads using the *Nextflow* (v22.10.2 (Di Tommaso et al. 2017)) workflow *nf‐polish* (available at https://github.com/MozesBlom/nf‐polish). The pipeline encompasses various read filtering procedures such as deduplication, trimming based on adapters and quality, merging reads and removing low‐complexity sequences. The polished reads were subsequently aligned to our de novo assembled *Melidectes torquatus* reference genome utilising *nf‐μmap* (*Nextflow* v23.10.1, accessible at https://github.com/IngoMue/nf‐umap) with *bwa‐mem2* being the chosen mapping algorithm (Müller 2022; Vasimuddin et al. 2019). The pipeline produces a summary report on mapping statistics and analyses common damage patterns observed in historical DNA (Vasimuddin et al. 2019). After mapping, we removed repetitive regions from the genomes using the previously generated .gff file as a mask and *BEDtools* (v2.31.1, (Quinlan and Hall 2010)).

### 2.3 Phylogenetic Analyses

We assembled mitochondrial genomes with *nf_mito‐mania* (*Nextflow* v22.10.2, available at: https://github.com/FilipThorn/nf_mito‐mania), keeping its standard configurations using our polished reads as input and the mitochondrial assembly of the Helmeted Honeyeater ( *Lichenostomus melanops cassidix* , NCBI accession: NC_062733 (Robledo‐Ruiz et al. 2022)) as the initial reference. The embedded variant calling mechanism within this pipeline removes sites that either fall below a depth‐of‐coverage of 20× or exceed threefold the individual's average genome‐wide mitochondrial coverage. Furthermore, to inspect potential sample contamination, mitochondrial assemblies were subjected to diploid variant calling. Given that mitochondria only exist as haploids, the presence of an excessive number of heterozygous sites would hint at possible cross‐sample contaminations.

The resulting consensus sequences from each specimen were aligned using *MAFFT* (v7.407, (Katoh and Standley 2013)). This alignment also included the Helmeted Honeyeater mitochondrion to serve as the outgroup. Occasionally, *Mitobim* produces artefacts in which some mitochondrial assemblies tend to be larger than expected when reads from the beginning of the mitochondria are being added to its end, due to the circular nature of mitochondria. These extended sections were clipped by trimming the alignments to the start and end position of the reference sequence through *seqtk* (v1.3, (Li 2024)) The identified start and end positions were additionally confirmed through manual examination with *AliView* (v1.28, (Larsson 2014)). This procedure ensured that the final sequence alignment was consistent and only contained regions of overlap (an aggregate length of 17,325 bp, including gaps). *Modeltest‐NG* (v0.1.7, (Darriba et al. 2020)) was run on the clipped alignment to determine the best substitution model (see Data S2 for parameters). The phylogenetic tree was obtained through *RAxML‐NG* (v1.1.0, (Kozlov et al. 2019)), by applying the GTR + I + G4 model of substitution as suggested by *modeltest‐NG*. Support values were generated through 100 bootstrapping iterations and a set of 10 random initial parsimony trees.

Using the same alignment, we additionally generated a dated mitochondrial phylogenetic tree by inferring divergence times between the species through *BEAST2* (v2.7.4, (Bouckaert et al. 2019)). We applied a GTR + G + I model, a Coalescent Bayesian Skyline tree model (Drummond et al. 2005) and a relaxed log normal clock model (Drummond et al. 2006) using a clock rate of 0.0205. The clock rate was obtained by calculating an average of the rates of all mitochondrial regions listed in the supporting information of Lerner et al. (2011). The MCMC chain was run for 10^8^ steps, using a burn‐in of 10^7^ steps and storing every 10^4^ steps to the log file. All detailed parameters implemented in *BEAST2* are given in Data S2. After verifying sufficiently high effective sample sizes (> 400) of each parameter through *Tracer* (v1.7.2, (Rambaut et al. 2018)), we produced a target tree through *treeannotator* and visualised it in *figtree* (v1.4.4, (Rambaut 2018)).

To generate a nuclear phylogenetic tree, we first performed individual variant calling on our bam files of individuals that had a depth‐of‐coverage (DoC) of at least 3× using *nf‐var* (https://github.com/MozesBlom/nf‐var). The pipeline was run on the largest 32 scaffolds which had the strongest similarities to chromosomes of the helmeted honeyeater genome. The pipeline was run on three subsets, (1) individuals below 10× DoC, (2) individuals between 10× and 25× and (3) one outlier individual with DoC 35.7×. Variants were called on an individual basis using *freebayes* (v1.3.6, (Garrison and Marth 2012)), low quality variants with allelic balances between 0 and 0.2 were removed and multiple nucleotide polymorphisms (MNPs) were deconstructed into SNPs. Afterwards, mask files containing filtered sites based on different criteria were generated. The applied filters removed insertions and deletions, heterozygous positions and sites with depth below 1/3 or above twice the average DoC within a subset. Lastly, the pipeline calculates missing data after filtering and produces consensus sequences for each individual and scaffold after applying all filters. For the final phylogeny, we only included individuals that had missing data < 40% (*n* = 100). The produced consensus sequences for these individuals were then used as input for *nf‐phylo* (https://github.com/MozesBlom/nf‐phylo), which generated window‐based maximum‐likelihood phylogenies for two window sizes (5000 and 10,000 bp), each sampled at 100 kb intervals. Using these window phylogenies, we used the pipeline to infer a summary coalescent phylogenetic tree through *ASTRAL* (v5.7.8, (Zhang et al. 2018)) and calculated site‐ and window‐concordance factors using *IQtree2* (v2.0.3, (Minh et al. 2020)).

The final mitochondrial and nuclear trees were visualised using *RStudio* (v2023.06.1 build 524, *R* version 4.2.3, (Posit team 2023; R Core Team 2021)) with the packages *tidyverse* (v2.0.0, (Wickham et al. 2019)) and *ggtree* (v3.12.0 (Yu et al. 2017)).

### 2.4 Population Structure

To quantify population substructure and to estimate levels of differentiation between samples, we used a genotype likelihood approach as implemented in *ANGSD* (v0.938) as this is better suited for low coverage data (Korneliussen et al. 2014). PCAs and Admixture were performed through *PCAngsd* (v0.982, (Meisner and Albrechtsen 2018)) and *NGSAdmix* (ngsadmix32, (Skotte et al. 2013)) respectively, both of which are implemented in the *Nextflow* workflow *nf‐GL_popstructure* (accessible at: https://github.com/FilipThorn/nf‐GL_popstructure). Admixture analyses were run with 10 replicates for each number of clusters (*K*) from which our individuals derive their ancestry. The full dataset with all species included (except for *M. torquatus* due to reference bias) was run from *K* = 1 to *K* = 10. A subset which only included individuals labelled as *M. belfordi* and *M. rufocrissalis* was run from *K* = 1 to *K* = 5. The results were plotted with custom *R* scripts through *RStudio* (v2023.06.1 build 524, R version 4.2.3) with the package *tidyverse* (v2.0.0). In order to determine best values of *K*, we first used a custom R script that applies the method described by Evanno et al. (2005), which considers the change of log likelihoods across each *K*. Additionally, we used *pong* (v1.5, (Behr et al. 2016)) which provides information on how many replicates converge on similar results and estimates average pairwise similarities across convergent runs.

### 2.5 Genetic Differentiation and Diversity

Estimates of genetic differentiation (*F*_ST_) and genetic diversity (through observed heterozygosity H_0_) were obtained through *ANGSD*. *F*_ST_ was calculated for different pairwise comparisons within *M. rufocrissalis* and *M. belfordi* populations but also between the species, including *M. foersteri* , *M. ochromelas* and *M. leucostephes* . Estimates were obtained in 100 kb windows with a 20 kb step size across the genome as well as genome‐wide using Hudson's estimator as presented in Bhatia et al. (2013).

Individual heterozygosity was estimated through *nf‐Hestu* (https://github.com/IngoMue/nf‐Hestu). This pipeline uses *ANGSD* to generate individual site frequency spectra (SFS) which allow for a direct estimation of observed heterozygosity. This pipeline removes sites below 1/3 and above twice an individual's average DoC.

### 2.6 Correlating Genetic Variation to Climatic Factors

First, we produced joint variant calls for a subset of *M. belfordi* and *M. rufocrissalis* (*n* = 72) in which they were treated as a single population using *freebayes*. Sites were subsequently filtered by among others removing sites with minor allele frequencies below 0.03, allowing only for a maximum of 20% missing data, Phred scores above 20, and a minimum as well as maximum depth per individual (min: 3× and max: 50×) and per variant (min: 6× and max: 48×). Additionally, multi‐nucleotide polymorphisms (MNPs) were decomposed into SNPs, and indels were removed.

We use gradient forest analysis with the R package *gradientForest* (GF) (Ellis et al. 2012) to test which climate variables explain the observed genetic variation best. For GF modelling, we used 500 regression trees to build a function for each SNP for each climatic variable. Only SNPs with *R* ^2^ > 0 (measure of response of individual SNPs to environmental gradients) were considered predictive loci and were further used in the aggregate turnover functions, accounting for the importance of climatic variables and the goodness of fit for each SNP. For the analysis, we used the 50 k random SNPs drawn from the previously generated vcf‐file filtered to consist only of variants with a sequencing quality of 30 and observed in at least 90% of all individuals. We also excluded all SNPs with a minimum allele frequency of 5% to avoid giving too much importance to rare alleles when looking for loci associated with environmental variation. A detailed description of the methodology and parameters is provided in Data S2; in most parts, we followed the same settings as Chen et al. (2022).

We predicted genome‐estimated breeding values (GEBVs) using the R package *rrBLUP* (Endelman 2011). GEBVs can be interpreted as predictions of ‘latent climate‐adapted phenotypes’, that is, unobserved phenotypes assumed to represent local adaptation to particular climate conditions (Gienapp et al. 2017; Lasky et al. 2015). We fitted genome–climate models and predicted climate‐associated phenotypes using isothermality (bio3), mean temperature of the driest quarter (bio9), and precipitation of the wettest month (bio13) to construct a multivariable analysis. We identified SNPs showing strong associations with the environmental variables identified in the gradient forest analysis based on a latent factor mixed‐effect model (LFMM) (Frichot et al. 2013).

The gradient forest analysis was extended to investigate in which part of the geographic distribution the individuals might be most vulnerable to climate change using an extension of the gradient forest analysis as described in Fitzpatrick and Keller (2015). The genomic offset is measured as the mismatch between current and predicted future genomic variation based on genotype–environment associations modelled across the contemporary distribution range. Populations with the greatest mismatch are least likely to adapt quickly enough to track future climate shifts, which potentially can lead to shifts in species ranges, population decline, or even extinction. To measure this, we harvested current and projected future values for each of 19 climatic variables from the 6237 2.5 arcmin grid cells across the combined distribution range. We downloaded current (1960–1990) and future climate data from the *WorldClim* database (v2.1, www.worldclim.org). To represent future climate scenarios, we used one CMIP6 future climate projection (MPI‐ESM1‐2‐HR) with two different emission scenarios (SSP 126 and SSP 585) for 2061–2070. SSP 585 represents the worst‐case scenario with an increased mean temperature of 3.5°C by 2070, while SSP 126 is a more optimistic scenario with an increase of 2.2°C by 2070. For each grid cell, climatic variables from both current and predicted climates were transformed based on the importance in predicting genomic variation. As a measure of genomic offset, we calculated the Euclidean distance between current and projected future values for each of the 6237 2.5 arcmin grid cells. Statistical differences in genomic offset between populations were tested with 10,000 permutations to estimate *p*‐values.

### 2.7 Vocal Differentiation

We sourced 25 song recordings of *M. belfordi* (19 individuals), 14 recordings of *M. rufocrissalis* (14 individuals) and 9 recordings of *M. torquatus* (7 individuals) from *Xeno‐canto* (https://xeno‐canto.org), an online repository of bird vocalisations. The song recordings were made across New Guinea between 1993 and 2019. All songs were visualised as spectrograms in the software *Luscinia* (Lachlan 2014), where we selected an average of 18 syllables—the constituents of a song per individual for acoustic analyses. Using the inbuilt dynamic time warping (DTW) algorithm with settings used in Rajan et al. (2024), we estimated the acoustic distance matrix between pairs of syllables using various acoustic features (time, mean frequency, change in mean frequency, etc.). Afterwards, we transformed the output distance matrix into Euclidean dimensions using non‐metric multidimensional scaling and thereafter a principal component analysis (PCA). The ordination of syllables in this PC space, thus represents overall acoustic dissimilarity observed across syllables of the three species.

We aggregated PCA scores of syllables per individual and used linear models to assess differences in the first and second principal components among the three focus species in the genus *Melidectes*. We included recording years as a covariate to account for temporal changes in songs due to stochastic and/or cultural changes over the 26‐year recording period. Post hoc tests with Tukey correction were performed using the *emmeans* package (v1.7.4, (Lenth 2024)). All statistical analyses were performed in R (v4.2.0).

## 3 Results

Our newly assembled reference genome for *Melidectes torquatus* had a total size of 1043 Mb spread over 911 scaffolds. As a comparison, the size of the Helmeted Honeyeater ( *Lichenostomus melanops cassidix* ) genome is 1103 Mb. The scaffold N50 of our genome was 71.3 Mb (L50 = 5), and the scaffold N90 was 11.6 Mb. BUSCO scores revealed that out of 62 BUSCO groups from 8338 avian genomes, 97% (8087) were complete and single‐copy, 0.4% (34) were complete and duplicate. 2.6% of all BUSCOs were fragmented (93) or missing (178). BUSCO scores were almost identical for the subset of our genome which only contained the largest 41 scaffolds that also had strong associations with chromosomes from other complete avian assemblies (C:97.3% [S:97.0%, D:0.3%], F:0.5%, M:2.2%).

Our evaluation of mapped reads against the *Melidectes torquatus* genome showed a mean depth‐of‐coverage (DoC) of ~9.51× (median: 7.03×, min: 1.24×, max: 36.71×, SD: 6.57) after masking for repetitive regions. See DoC for each individual in Table S4. Two individuals (all libraries for one individual, one library for the second) showed an elevated number of heterozygote sites (> 50) spread evenly across their mitochondria, indicating possible contamination. Since we are interested in investigating hybrids in this study, we decided to remove one sample and the potentially contaminated library of the second individual from subsequent analyses as they could yield false signals of hybridisation.

### 3.1 Phylogenetic Analyses

**FIGURE 2:**
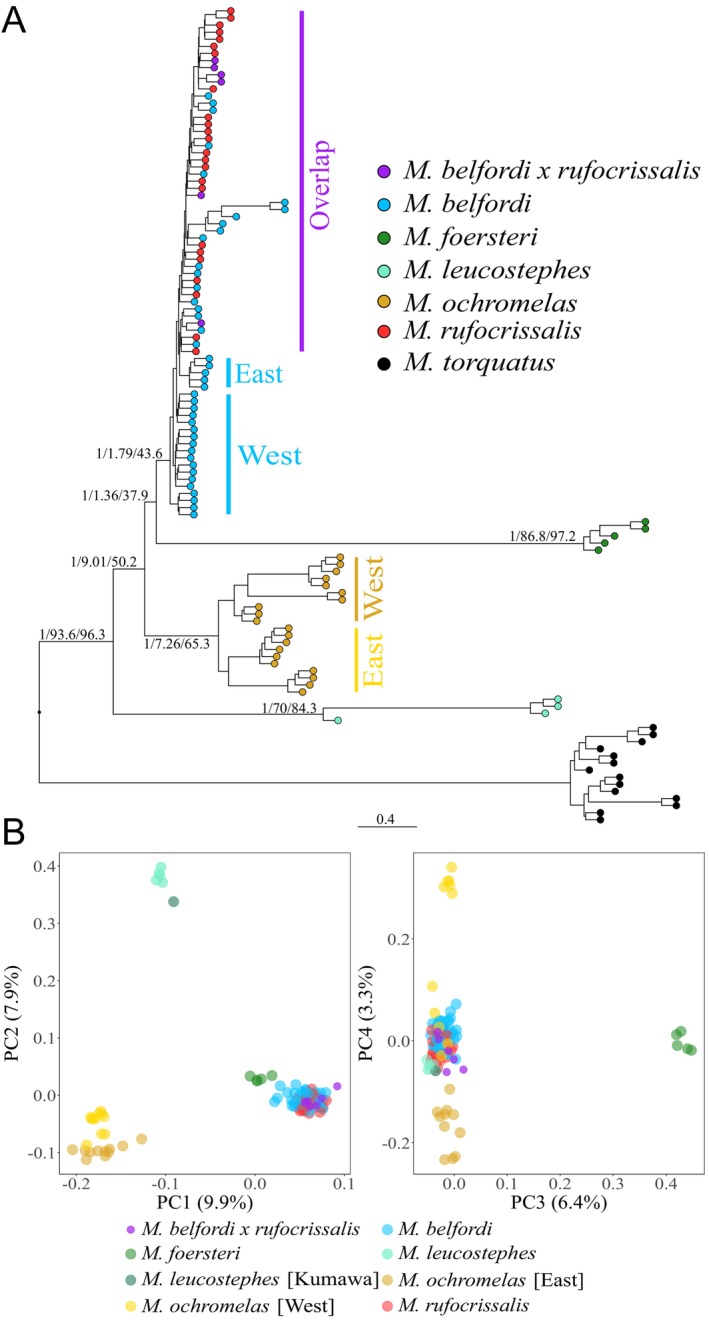
(A) Autosomal phylogenetic tree (summary coalescent based on 10‐kbp windows) for Melidectes, midpoint‐rooted with *M. torquatus* as the outgroup. Support values for selected clades describe bootstraps/window concordance factors/site concordance factors. Western and eastern populations of *M. ochromelas* and *M. belfordi* , as well as individuals from the overlapping distribution of *M. belfordi* /rufocrissalis, are labelled additionally. (B) PCA based on autosomal loci for all species except for *M. torquatus* . Colours represent different species and subpopulations of *M. ochromelas* and *M. leucostephes* .

Our mitochondrial (Figure S7) and autosomal phylogenetic trees (Figure 2A) support the same basal species relationships ( *M. torquatus* , *M. leucostephes* , *M. ochromelas* ) as previously described in Marki et al. (2017) and placed *M. foersteri* as sister to the *M. belfordi* and *M. rufocrissalis* group, albeit with poor support. Several long branches between species indicate strong genetic divergences at the species level. In the mitochondrial phylogenetic tree (Figure S7), groups within *M. torquatus* corresponded only weakly to geographic proximity as western and eastern populations were placed in the same clade. However, some populations such as one from the Bird's Head peninsula (*M. t. torquatus*) and populations from the very east of the island, that is, the Huon and southeastern Papuan Peninsula were grouped in clades (see Figure S1). The autosomal phylogenetic tree (Figure 2A) produced a similar topology although most populations formed monophyletic groups except for *M. torquatus polyphonus* . The populations from the Bird's Head and Huon peninsula also showed long branch lengths suggesting stronger isolation in these regions. Most subdivisions of *M. leucostephes* and *M. ochromelas* were separating subspecies and populations inhabiting distinct montane areas in both phylogenies (Figures S2, S3 and S7, Figure 2A). We note a very deep divergence of one individual of *M. leucostephes* sampled in the Kumawa mountains from its conspecifics on the Bird's Head (Figure S2). Relationships within *M. ochromelas* fit according to its recognised subspecies with the exception of *M. ochromelas batesi* individuals from the Weyland mountains in western New Guinea that were closer related to *M. o. ochromelas* that occur in the northwest of the island than to individuals from their own subspecies that inhabit the southeast (Figure S3). The main split within this species divided populations from the western and eastern end of the island. *Melidectes foersteri* and *M. leucostephes* , which both have more restricted distributions compared to the other species, both exhibited very long branch lengths in the autosomal phylogenetic tree (Figure 2A), suggesting the accumulation of many shared mutations in isolation within each species.

*Melidectes belfordi* and *M. rufocrissalis* did not form monophyletic groups and are mixed throughout a clade. Only the westernmost populations (often considered as subspecies *M. belfordi kinneari* , *M. belfordi joiceyi* and *M. belfordi griseirostris*; individuals labelled as ‘West’ in Figure 2A, Figures S7, S8, S11 and S12) formed a non‐admixed *M. belfordi* clade (Figures S6 and S7). In the autosomal phylogenetic tree, these populations diverge sequentially from the westernmost population towards the east (basal clades of the *M. belfordi* /*rufocrissalis* clade in Figure 2). The only other two monophyletic groups within the *M. belfordi* /*rufocrissalis* complex were *M. belfordi* from the eastern end of the distribution, specifically the Papuan Peninsula and one locality near Mt. Herzog in the Eastern Ranges (Figures S6 and S7).

**FIGURE 3:**
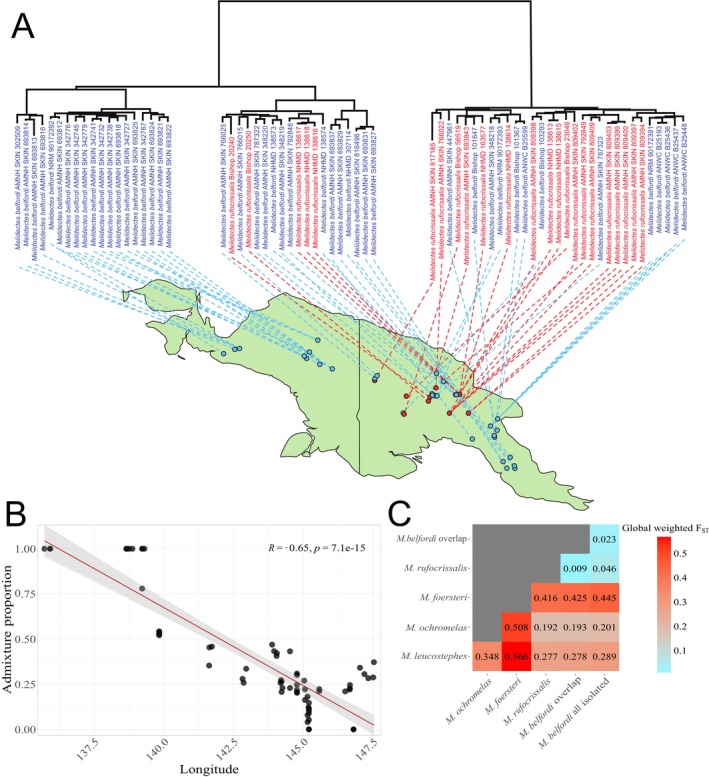
(A) Pruned mitochondrial phylogenetic tree of the *M. belfordi* and *M. rufocrissalis* clade. Individuals are projected onto a map to show their sampling site. (B) Admixture proportion of *M. belfordi* and *M. rufocrissalis* individuals at *K* = 2 across a longitudinal west to east gradient (proportion shown for the blue ancestral cluster in Figures S12 and S13, i.e., the ancestral cluster, which is most prevalent in *M. belfordi* from western populations [labelled West in Figure 2A]). Kendall's rank correlation coefficient and its *p*‐value were measured through the R package ggpubr (Kassambara 2023). (C) Global weighted *F*_ST_ for *M. belfordi* and *M. rufocrissalis* and the basal sister species. *M. belfordi* is further subdivided into populations where only *M. belfordi* occurs (‘all isolated’) and where *M. belfordi* overlaps with the distribution of *M. rufocrissalis* (‘overlap’).

The mitochondrial phylogenetic tree showed a different topology (Figure S7) with one deep split within *M. belfordi* */rufocrissalis* at about the same time as *M. foersteri* diverged from this complex. One clade included not only all *M. belfordi* from outside the overlapping distribution with *M. rufocrissalis* at both the western and eastern ends but also some individuals of *M. rufocrissalis* (Figure 3A). The second main lineage was more mixed but included more individuals labelled as *M. rufocrissalis* . Individuals that were identified as hybrids were found in both main lineages. In both phylogenies, populations from within regions where both taxa occur no longer follow recognised species boundaries and instead were grouping by geographic proximity of the individuals' sampling location. Mitochondrial differentiation within taxa was low, as exhibited by the generally short branch lengths (Figure S7).

The divergence times we obtained through *BEAST2* (Figure S8) estimated the first split of *M. torquatus* to the remaining species at 4 mya (95% highest probability density [HPD]: 3.2–5 mya). *Melidectes leucostephes* diverged about 2.7 mya (95% HPD: 2.2–3.4 mya). Divergence times of *M. ochromelas* (1.3 mya, 95% HPD: 1.1–1.5 mya), *M. foersteri* (1.2 mya, 95% HPD: 1–1.4 mya) and the deep split within *M. belfordi* + *rufocrissalis* (1.2 mya, 95% HPD: 0.9–1.4 mya) were much closer to each other, with largely overlapping credible intervals.

### 3.2 Population Structure

Results from PCAs (Figure 2B) and admixture analyses (Figure S10) were similar to the mitochondrial phylogenetic tree. Most species ( *M. foersteri* , *M. ochromelas* , *M. leucostephes* ) show up as clear and distinct clusters in both PCA as well as admixture analyses and do not provide much evidence of admixture between the species (Figure S10), one notable exception being the seemingly asymmetric gene flow from *M. foersteri* into *M. belfordi* */rufocrissalis*. Evaluations of best *K* using the method described by Evanno et al. (2005) identified *K* = 2 as best for the dataset including all species except for *M. torquatus*, but *K* = 3 for a subset of this data which only included *M. belfordi* and *M. rufocrissalis* . Our analysis using *pong* showed that results converged at *K* = 3 (7/10 runs, average pairwise similarity = 0.998) and *K* = 5 (8/10 runs, average pairwise similarity = 0.997) for the genus dataset and at *K* = 3 (10/10 runs, average pairwise similarity = 0.988) for the subset of *M. belfordi* and *M. rufocrissalis* . Unfortunately, neither method is suitable to test whether *K* = 1 would be best. When looking at higher principal components (PC1 to PC8, Figure S9) *M. belfordi* and *M. rufocrissalis* are never clearly separated as a whole group. At *K* = 5, *M. ochromelas* is subdivided similarly between eastern and western individuals, but also individuals with mixed proportions of both ancestries, yet *M. belfordi* */rufocrissalis* share the same ancestry. At *K* = 6, a second ancestry appears within the *M. belfordi* */rufocrissalis* complex, but proportions are highly mixed in most populations except for *M. belfordi* individuals occurring outside the overlapping distribution and some but not all individuals of *M. rufocrissalis* from one locality (Denge Numbu). To investigate this further, we reran the same analyses focusing just on *M. belfordi* */rufocrissalis*.

Our PCA (Figure S11) did not separate *M. belfordi* from *M. rufocrissalis* when individuals were from the overlapping distribution of the two taxa. Only *M. belfordi* individuals from outside the overlapping distribution and the Lae population (Figure S12, leftmost individuals of ‘ *M. belfordi* (Overlap)’ on PC1) formed distinct groups.

At *K* = 2 (Figures S12 and S13A), individuals that were fixed for both ancestries were mainly found within *M. belfordi* ; only two individuals of *M. rufocrissalis* showed no signal of admixture (again from Denge Numbu). Admixture proportions instead changed from one ancestry to another almost gradually as one moves from west to east (Figure 3B), regardless of species identity. At higher values of *K* (i.e., 3 and 4) non‐overlapping populations were shown with mostly fixed proportions of the newly added ancestry (Figures S12 and S13B,C). The distribution of the two taxa and mean admixture proportions of populations are shown as an overview in Figure S13.

### 3.3 Genetic Differentiation and Diversity

Observed heterozygosity *H*_0_ values (Table S5, Figure S14) showed a pattern in which, compared to other species in the genus, *M. belfordi* and *M. rufocrissalis* had much higher (on average up to threefold) values. Estimates for *M. belfordi* from within the overlapping region (*H*_0_ = 0.0031) did not differ significantly from *M. rufocrissalis* (*H*_0_ = 0.0030). *Melidectes foersteri* (*H*_0_ = 0.0010) and *M. leucostephes* (*H*_0_ = 0.0011) showed the lowest values within the genus and similar levels to each other. *M. ochromelas* exhibited intermediate estimates (*H*_0_ = 0.0018).

Genome‐wide *F*_ST_ estimates range from 0.009 to 0.477 depending on the pairing (Figure S15). The lowest value of differentiation was found between overlapping *M. belfordi* and *M. rufocrissalis* (Figure 3C), while the highest values were obtained in any pairing against *M. foersteri* . *M. belfordi* populations outside the overlap exhibited lower differentiation to *M. belfordi* from within the overlap than to *M. rufocrissalis* (roughly twice as high *F*_ST_).

### 3.4 Correlating Genetic Variation to Climatic Factors

Environmental variables that were most strongly associated with genetic variation within the *M. belfordi* */* *rufocrissalis* clade were precipitation, temperature and isothermality. Estimates of genomic offset (how much a population has to change genetically in response to future climate change) showed that across most of their distribution, *M. belfordi* + *M. rufocrissalis* exhibited generally low estimates, but western populations outside the overlap showed higher values of genomic offset.

The gradient forest analysis identified three environmental variables (of the 19 variables tested) that explained ca. 60% of the observed genetic variation across the combined distribution of *M. belfordi* and *M. rufocrissalis* . These top three explanatory variables were isothermality (bio3), mean temperature of the driest quarter (bio9) and precipitation of the wettest month (bio13). Precipitation in the wettest month (bio13) was the most important predictor of the genetic response to the environmental conditions in the central region of the Central Range (yellowish colours in Figure S19). Variation in the combined effects of isothermality (bio3) and mean temperature of the driest quarter (bio9) was most responsible for the genetic structure of individuals occurring in the western and eastern parts of the distribution range (Figure S19).

To detect local adaptation to a given climate condition, we calculated genome‐estimated breeding values (GEBVs) as predictions of ‘latent climate‐adapted phenotypes’ using *rrBLUP* (Endelman 2011; Gienapp et al. 2017; Lasky et al. 2015). We found that GEBVs vary along the gradients of our three tested climatic variables, reflecting adaptation of the climate latent phenotypes to the different climate conditions, that is, local adaptation (Figure S20).

Using the latent factor mixed model (LFMM) (Frichot et al. 2013) we identified a total of 23 SNPs that were found to be significantly associated with bio3, 25 with bio9 and five with bio13.

**FIGURE 4:**
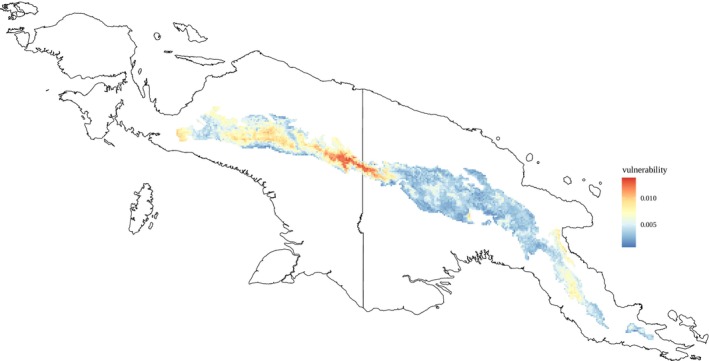
Local genetic offset modelling using Gradient Forest analyses of current and predicted future climate conditions. The genetic offset is a measure of how much genetic change is needed for a population to adjust to new climate conditions by local adaptation. Results based on the worst‐case scenario 2070 SSP 585. Populations in the northwestern parts of the distribution range are most vulnerable to the predicted climate change. Additionally, the population in the central region of the Central Range (shown in darkest red, in the area between ‘Mount Yamin’ and ‘Central Range’ labels in Figure S6) will exhibit extreme environmental stress.

On the basis of these climate‐associated genotypes, we predicted which populations might be most vulnerable to climate change. We calculated local genetic offset as a measure of how much genetic change is needed for a population to adjust to new climate conditions by local adaptation (Fitzpatrick and Keller 2015), that is, without dispersal. We calculated the genetic offset for two emission scenarios for 2061–2070, an upper‐boundary scenario (SSP 585) leading to an increased mean temperature of 3.5°C by 2070, and a more moderate scenario that assumes undertaken climate protection actions (SSP 126) with an increase of 2.2°C by 2070. We found that under both scenarios the populations in the northwestern parts of the distribution range are the most maladapted to the predicted climate change (Figure 4, Figure S21). Under the more severe scenario, the population in the central region of the Central Range (darkest red region in Figure 4, in the area between the ‘Mount Yamin’ and ‘Central Range’ labels in Figure S6) could be exposed to extreme environmental stress. The populations in the eastern part of the Central Range (Papuan side of New Guinea where *M. belfordi* and *M. rufocrissalis* overlap in their distributions) are predicted to be less affected by the climate change under both scenarios.

### 3.5 Vocal Differentiation

The first two principal components together explained nearly half (45.68%) of the variance in syllables (constituents of songs) across the three species: *M. belfordi* , *M. rufocrissalis* and *M. torquatus* . We did not find any significant differences among individuals of the three species in the first principal component (Figure S16, linear regression, Sum sq. = 0.001, df = 2, *F* = 0.196, *p* = 0.822). However, the species differed significantly in the second principal component (Figure S16, linear regression, Sum sq. = 0.0144, df = 2, *F* = 4.798, *p* = 0.013). Post hoc tests revealed that *M. torquatus* individuals have songs that are distinguishable from those of *M. belfordi* (estimate = 0.045, SE = 0.015, *t*.ratio = 3.013, *p* = 0.011) and *M. rufocrissalis* (estimate = 0.042, SE = 0.016, *t*.ratio = 2.542, *p* = 0.038). In contrast, there were no significant differences between the songs of *M. belfordi* and *M. rufocrissalis* individuals (estimate = 0.002, SE = 0.013, *t*.ratio = 0.203, *p* = 0.977). Lastly, the year of recording had no significant effect on either of the two principal component scores (PC1: *p* = 0.61, PC2: *p* = 0.51), suggesting that there was minimal variation in the songs over the years.

These results indicate that while songs across the genus *Melidectes* are rather similar, *M. torquatus* has a distinct acoustic profile that sets it apart from the other species. On the other hand, *M. belfordi* and *M. rufocrissalis* exhibit much greater acoustic similarity, and their songs cannot be reliably distinguished based on the first two principal components.

## 4 Discussion

Using whole‐genome resequencing data from 124 individuals across the entire distributional range of New Guinea for all six currently recognised species of *Melidectes*, we find evidence of allopatric speciation in isolated montane regions, but also evidence for segregation along elevational gradients. Furthermore, we show that species/populations with small and isolated distributional ranges exhibit low genetic diversity and high genetic differentiation compared to species/populations with large and widespread distributional ranges. Most notably, we reveal one case of ephemeral speciation, where two lineages started to speciate but then fused back into a single lineage. Finally, using climatic niche modelling, we discuss how climate may shape future speciation events within the clade.

### 4.1 Allopatric Speciation in *Melidectes*

Analyses of both the nuclear and the mitochondrial data demonstrate that four members of *Melidectes* ( *M. foersteri* , *M. leucostephes* , *M. ochromelas* and *M. torquatus* ) are reciprocally monophyletic (Figure 2 and Figure S7), whereas *M. belfordi* and *M. rufocrissalis* are mixed. The phylogenetic relationships generally reflect biogeographical expectations, that is, speciation has largely taken place in allopatry when populations in isolated outlying mountain ranges have become geographically isolated from the large Central Mountain Range that traverses New Guinea from west to east (Mayr and Diamond 2001; Pujolar et al. 2022; Stelbrink et al. 2022). For example, *M. foersteri* , restricted to the isolated Huon mountains (Figure S4), is sister to the *M. belfordi/rufocrissalis* complex from the large Central Range (Figure 2, Figures S5 and S6). The general explanation for this common pattern is that populations occupying these two regions were largely connected in the past either when the mountains were lower or during colder times when montane forest expanded down‐slope. However, at present, the extensive lowland valley that separates the two mountain regions represents a strong barrier for gene flow for many vertebrate species in the region (Wikramanayake et al. 2002). We find no sign of gene flow from *M. belfordi/rufocrissalis* into *M. foersteri* , but some gene flow in the opposite direction (Figure S10). This goes against the general biogeographical expectation that the Central Mountain Range serves as a source for colonisation of the outlying ranges but confirms observations from other avian taxa in New Guinea (Pujolar et al. 2022).

For *M. ochromelas* we recover a deep split (noticeable in PC4 and distinct ancestries at *K* = 5 (Figure 2B and Figure S7)) between the eastern and the western populations (notice that this taxon is absent in central New Guinea, see Figure S3). Further subdivisions separate populations of the outlying Huon peninsula from eastern populations as well as populations from the Bird's Head and Bird's Neck from central populations. A similar pattern is observed for *M. leucostephes* , which is found on the Bird's Head and the Bomberai peninsula with almost no elevational overlap with other congeners (Figure S2). As for *M. foersteri* , the branch leading to this species is long (Figure 2A) and comparatively higher levels of differentiation (*F*_ST_) indicate that this species is well‐separated from its congeners (Figure 3 and Figure S7). The low levels of heterozygosity (Table S5 and Figure S14) could also be the result of a small population size within this species, further supporting the long branch lengths due to the faster accumulation of substitutions driven by genetic drift. One individual from the Kumawa mountains is well separated from populations on the Bird's Head. This population could be considered a separate valid species and represents another example of (incipient) allopatric speciation. *Melidectes torquatus* occurs across the entire Central Range as well as in isolated mountains on the Bird's Head and the Huon Peninsula. The continuous distribution is likely the reason that we find low levels of differentiation within the species. Furthermore, this species inhabits lower elevations than its congeners, likely allowing for more continuous mixing of populations as seen in other avian taxa across New Guinea (Pujolar et al. 2022).

### 4.2 A Case of Ephemeral Speciation

Based on distribution patterns and the observation of phenotypic hybrids between *M. belfordi* and *M. rufocrissalis* , Mayr and Gilliard (1952) formulated a hypothesis of how these species had formed, dispersed and come to hybridise. Specifically, Mayr and Gilliard (1952) proposed that the two species had formed in allopatry and that upon secondary contact they were differentially adapted to high and low elevations, respectively. Based on previous work (Mayr and Gilliard 1952) and personal field experience (KAJ), it is known that morphological hybrids are common at elevations where the two species co‐occur and become successively scarcer towards elevations where only one species is found. Mayr and Gilliard (1952) considered this the result of a selective advantage of wattles ( *M. rufocrissalis* ) in open forests at lower elevations while black bills ( *M. belfordi* ) were positively selected for in dense forest at higher elevations. All our analyses show that samples morphologically assigned to *M. belfordi* and *M. rufocrissalis* are mixed (Figures 2 and 3) and that there is no genetic signature of elevational replacements ( *M. belfordi* above *M. rufocrissalis* , Figure S17), as individuals occurring at the highest and the lowest elevations tend to be equally admixed as individuals at the proposed elevational overlap. Instead, we find the lowest levels of admixture in individuals occurring in the western and eastern ends of the distribution. This corresponds to areas of New Guinea where only individuals morphologically identified as *M. belfordi* occur (Figure S13). Furthermore, we are not able to detect any distinguishable differentiation in vocalisation between *M. belfordi* and *M. rufocrissalis* (Figure S16) suggesting the lack of vocal pre‐mating isolation. These results provide no support for *M. belfordi* and *M. rufocrissalis* as being well‐differentiated species and should thus be treated as a single taxonomic unit.

The probability of hybridisation between recently diverged lineages that come into secondary contact is often high and although incipient species may be common, they are likely short‐lived in nature (ephemeral speciation) (Rosenblum et al. 2012). The literature is replete with possible cases of lineage fusions or even speciation reversals (Behm et al. 2010; Block et al. 2015; Kearns et al. 2018; Taylor et al. 2006), but unambiguous examples remain scarce. Our genomic analyses suggested that the *M. belfordi/rufocrissalis* complex constitutes such an example. First, our analyses of the nuclear genomes of *M. belfordi* and *M. rufocrissalis* densely sampled across their distributional range provide no support for two genetically separated units today (Figures 2 and 3). The levels of divergence and introgression instead follow a clear geographical pattern in which populations in the westernmost and eastern‐most parts of their distribution (where only *M. belfordi* occurs) are most divergent and least introgressed. This pattern is also evident from the admixture analysis for *K* = 2 in which admixture proportions are correlated with geographic distance (irrespective of the species assignment, Figure 3B), which is expected to be a result of isolation by distance within a single species. However, *F*_ST_ and heterozygosity estimates do support a recent fusion of two lineages into one. Individuals of *M. belfordi* from outside the region where *M. belfordi* and *M. rufocrissalis* co‐occur are less differentiated from individuals labelled as *M. belfordi* from within the area of co‐occurrence (Figure 3C, *F*_ST_ = 2.3%) than with individuals of *M. rufocrissalis* (Figure 3C, *F*_ST_ = 4.6%). As we consider isolated populations of *M. belfordi* to carry ancestral haplotypes, these results indicate that *M. belfordi* used to be more differentiated from *M. rufocrissalis* . The comparatively high values of observed heterozygosity in overlapping populations of the *M. belfordi/rufocrissalis* complex compared to other species of *Melidectes* ( *M. ochromelas* , *M. foersteri* and *M. leucostephes* ) are likely the outcome of extensive mixing of two genetically distinct lineages (Table S5).

The mitochondrial tree (Figure 3A, Figure S7) differs significantly from the autosomal tree, showing a deep split within the *M. belfordi/rufocrissalis* complex that is almost as deep as the split to their two most closely related species, *M. foersteri* and *M. ochromelas* . Moreover, these two mitochondrial clades agree better with the currently recognised species delimitation, as one of the clades consists mostly of individuals morphologically identified as *M. belfordi* (31 out of 38) whereas the other clade consists mainly of individuals morphologically identified as *M. rufocrissalis* (18 out of 31). Significantly, however, *M. belfordi* from outside the area of overlap, at the western and eastern ends of New Guinea, only has mitochondria from the predominantly *M. belfordi* lineage. This suggests that *M. belfordi* once only carried mitochondria from this lineage and that the other mitochondrial lineage originated from a differentiated *M. rufocrissalis* .

Alternatively, one of the two divergent mitochondrial lineages found within the *M. belfordi/rufocrissalis* complex is a result of mitochondrial capture (e.g., Andersen et al. 2021) from a founder population. However, such a scenario seems unlikely as the closest relatives ( *M. foersteri* and *M. ochromelas* ) have even more divergent mitochondrial lineages and as the most eastern and western populations both carry mitochondria of the ‘ *M. belfordi* ’ type. The observed lack of a clear structure and high admixture between the individuals assessed as *M. belfordi* and *M. rufocrissalis* does not necessarily imply that there are no barriers to gene flow between certain populations within this complex. Single large‐effect loci may still be present that maintain barriers between other not currently recognised populations as observed, for example, in *Heliconius* butterflies or crows (Martin et al. 2013; Poelstra et al. 2014). Such loci can involve, for example, single genes responsible for various pre‐ or postzygotic isolation mechanisms or their regulatory elements, but also structural variants that can act as reproductive barriers, such as chromosomal inversions (Faria et al. 2019; Martin et al. 2013; Poelstra et al. 2014; Powell et al. 2020). However, through our window‐based *F*_ST_ scans (Supporting Information), we do not identify significant peaks unique to a pairwise comparison within *M. belfordi/rufocrissalis* that would indicate regions which could potentially harbour loci responsible for maintaining reproductive barriers. Although the Z chromosome exhibits higher values of *F*_ST_ than autosomes, this pattern was consistent across all species comparisons and is again lowest when pairing *M. belfordi* from within the overlap against *M. rufocrissalis*.

While most of the nuclear data offer limited signals of the *M. belfordi/rufocrissalis* complex having been distinct species in the past (likely due to extensive mixing over substantial time), the mitochondrial pattern and our *F*_ST_ results collectively support a scenario of ephemeral speciation.

### 4.3 Future and Incipient Isolation

Information on movement behaviour in the *Melidectes* is scarce (but see Reeve et al. (2022) for real‐time movement data for some New Guinean bird species), yet most New Guinea bird species are presumed to be sedentary (Del Hoyo et al. 2008). A low movement capacity is often described as an important driver for strong differentiation and subsequently effective allopatric speciation (Sánchez‐Montes et al. 2018; Smith et al. 2014). Our study identifies two populations within the *M. belfordi* */rufocrissalis* complex that appear to be relatively more isolated across the genomes. The population at Mount Herzog, *M. belfordi stresemanni*, immediately south of the Huon peninsula (leftmost individuals within the *‘M. belfordi* (Overlap)’ group in Figure S12) appears to be non‐admixed at *K* = 2 and forms its own cluster at higher *K* values (yellow at *K* ≥ 4 in Figure S12). A similar pattern is evident at *K* = 4 for the southeastern population, which is sometimes referred to as the subspecies *M. belfordi brassi* (labelled ‘ *M. belfordi* (East)’ in Figure S12). These two populations are geographically relatively isolated and thus, may represent cases of parapatric incipient isolation, which in the future may lead to speciation if gene flow remains limited.

Given that speciation within montane taxa on New Guinea appears to frequently take place in allopatry and is largely driven by Pleistocenic climate fluctuations, anthropogenic global warming could potentially also lead to the isolation of populations and subsequently incipient speciation. The climatic analyses (gradient forest, Figure 4) suggest that populations of *M. belfordi* around Mount Yamin in the Border Ranges (immediately to the west of the region where *M. belfordi* */rufocrissalis* morphs occur) exhibit the highest levels of genomic offset (Figure 4), indicating that these populations may be most vulnerable to climate change as their genomes would need to undergo significant adaptational changes to withstand future climate change. If these populations were to disappear in the future and without significant dispersal, global warming may thus lead to isolation (and potentially speciation) of the western populations of *M. belfordi* from the eastern *M. belfordi* /*rufocrissalis* population. However, further analyses that investigate temporal patterns of genome erosion or population genetic simulations are needed to provide more accurate predictions regarding the future population dynamics of this species.

## Author Contributions

The study has been conceived and designed by K.A.J. and M.I. I.A.M. and M.I. carried out the laboratory work. R.‐A.O. and V.P. were involved in the de novo genome assembly. I.A.M. and F.T. carried out analyses on genetic data with input from K.A.J., M.I. and M.P.K.B. Additionally, S.R. performed analyses on vocal differentiation, and P.G.P.E. ran correlations of genetic data and climatic factors. I.A.M. drafted the manuscript with contributions from K.A.J., M.I., M.P.K.B., S.R. and P.G.P.E. I.A.M., F.T., S.R., R.‐A.O, P.G.P.E., V.P., B.T.S., G.M., B.K., B.I., M.P.K.B., M.I. and K.A.J. provided comments and feedback on the manuscript.

## Disclosure

Benefit‐Sharing: The majority of samples in this study are hosted in various natural history collections and have been collected during expeditions up to several decades ago. The Nagoya protocol is therefore not applicable for these samples. Fresh samples that are included in this study were collected in compliance with all relevant regulations. Required permits including research permits (99902749307 to K.A.J.) and export permits (017179 and 19069) have been obtained through the Conservation and Environment Protection Authority (CEPA) of Papua New Guinea.

## Conflicts of Interest

The authors declare no conflicts of interest.

## Supporting information


Data S1

**Table S4.** List of all samples included in this study. Information on museum vouchers, morphological species and sex identifications, sampling localities and years as well as depth‐of‐coverage estimates are provided. Codes for each museum collection are explained at the bottom of the sheet.

**Table S5.** Estimates of observed heterozygosity for all species (and morphological hybrids) excluding *M. torquatus* . Columns include means, standard deviations, medians, minima and maxima of individual estimates within each population.

**Data S2.** FST_plots.

## Data Availability

Raw sequencing data and metadata have been deposited at the European Nucleotide Archive (ENA) under project accession PRJEB83823. Additional metadata are included in Table S4.
