# Supplementary material for: Ephemeral Speciation in a New Guinean Honeyeater Complex (Aves: Melidectes)
Source: Mol Ecol. 2025 Apr 11;34(21):e17760. doi: 10.1111/mec.17760 (PMC12573753; doi:10.1111/mec.17760)
Supplement: Supplementary file 1 — Data S1 [file MEC-34-e17760-s004.docx]

**Supplementary Material**

**Title:**

**Ephemeral speciation in a New Guinean honeyeater complex (*Aves: Melidectes*)**

**Authors:**

Ingo A. Müller^1,2,3*^, Filip Thörn^1,2,3^, Samyuktha Rajan^2^, Remi-André Olsen^4^, Per G.P. Ericson^1^, Valentina Peona^1,5^, Brian Tilston Smith^6^, Gibson Maiah^7^, Bonny Koane^7^, Bulisa Iova^8^, Mozes P.K. Blom^3^, Martin Irestedt^1a^, Knud A. Jønsson^1a^

^1^ Department of Bioinformatics and Genetics, Swedish Museum of Natural History, Stockholm, Sweden

^2^ Department of Zoology Stockholm University, Sweden

^3^ Museum für Naturkunde, Leibniz Institut für Evolutions- und Biodiversitätsforschung, Berlin, Germany

^4^ Science for Life Laboratory, Department of Biochemistry and Biophysics, Stockholm University, Solna, Sweden

^5^ Swiss Ornithological Institute Vogelwarte, Sempach, Switzerland

^6^ Department of Ornithology, American Museum of Natural History, New York, USA

^7^ New Guinea Binatang Research Centre, Madang, Papua New Guinea

^8^ Papua New Guinea National Museum and Art Gallery, Port Moresby, Papua New Guinea

^a^ Shared last authors

^*^ Corresponding author: Ingo.Mueller94@gmail.com

**Contents**

[Supplementary tables 3](#_Toc186738205)

[Supplementary figures 4](#_Toc186738206)

[Supplementary methods 18](#_Toc186738207)

[Correlating genetic variation to climatic factors (expanded) 18](#_Toc186738208)

[Vocal differentiation (expanded) 28](#_Toc186738209)

[Codes and parameters 30](#_Toc186738210)

# Supplementary tables

**Table S1.** Github commits that were used for each tool and nextflow workflow

| Tool/Workflow | Github commit |
| --- | --- |
| YaHS | 42b8421115340ee7b9d52ff63e7f54d28084871c |
| nf-polish | 3b9aad33f40f7a4abc8c2ca109fb4c4072e187d6 |
| nf-µmap | 676f5c420f196fd49d3ecf6969d73e027207364e |
| nf_mito-mania | 06925a620523edd4480af65a10db11e00e2394a3 |
| nf-GL_popstructure | 496f4a47557857e35b1a43632122535bf4156686 |
| nf-Hestu | fb8d16c35025db9de5fdf32aa9c87596fd219a23 |
| nf-var | 1d860e76798d14c0600b9a8aceb2fc4699689fc9 |
| nf-phylo | ffc4bb12088a364129915ade7e07073879c6f9b7 |

# Supplementary figures


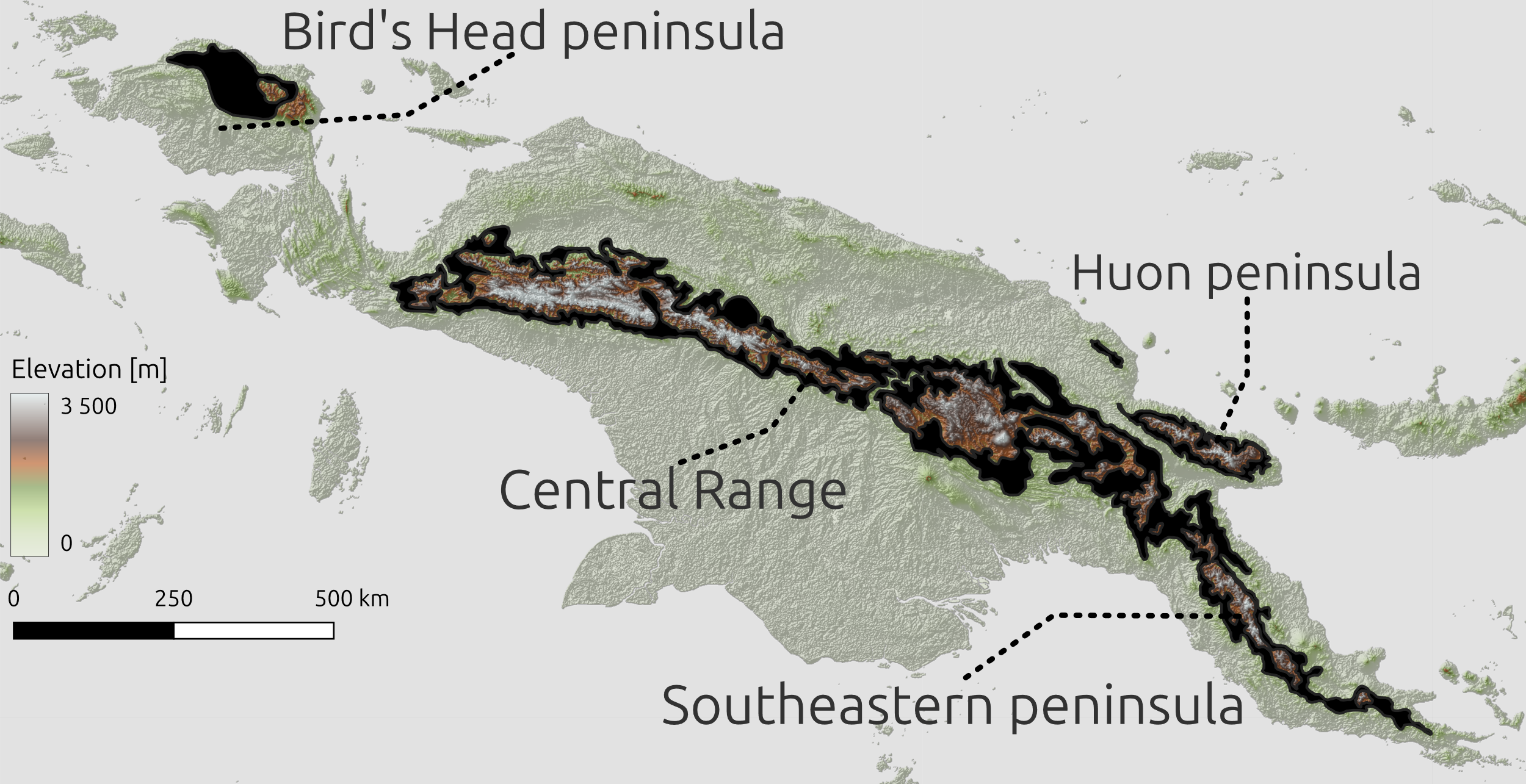


Figure S1. Distribution map of Melidectes torquatus. Relevant regions from results and discussion are labelled on the map. Distributional data obtained from the IUCN red list (originally hosted by BirdLife). Topographic data was collected from the United States Geological Survey.


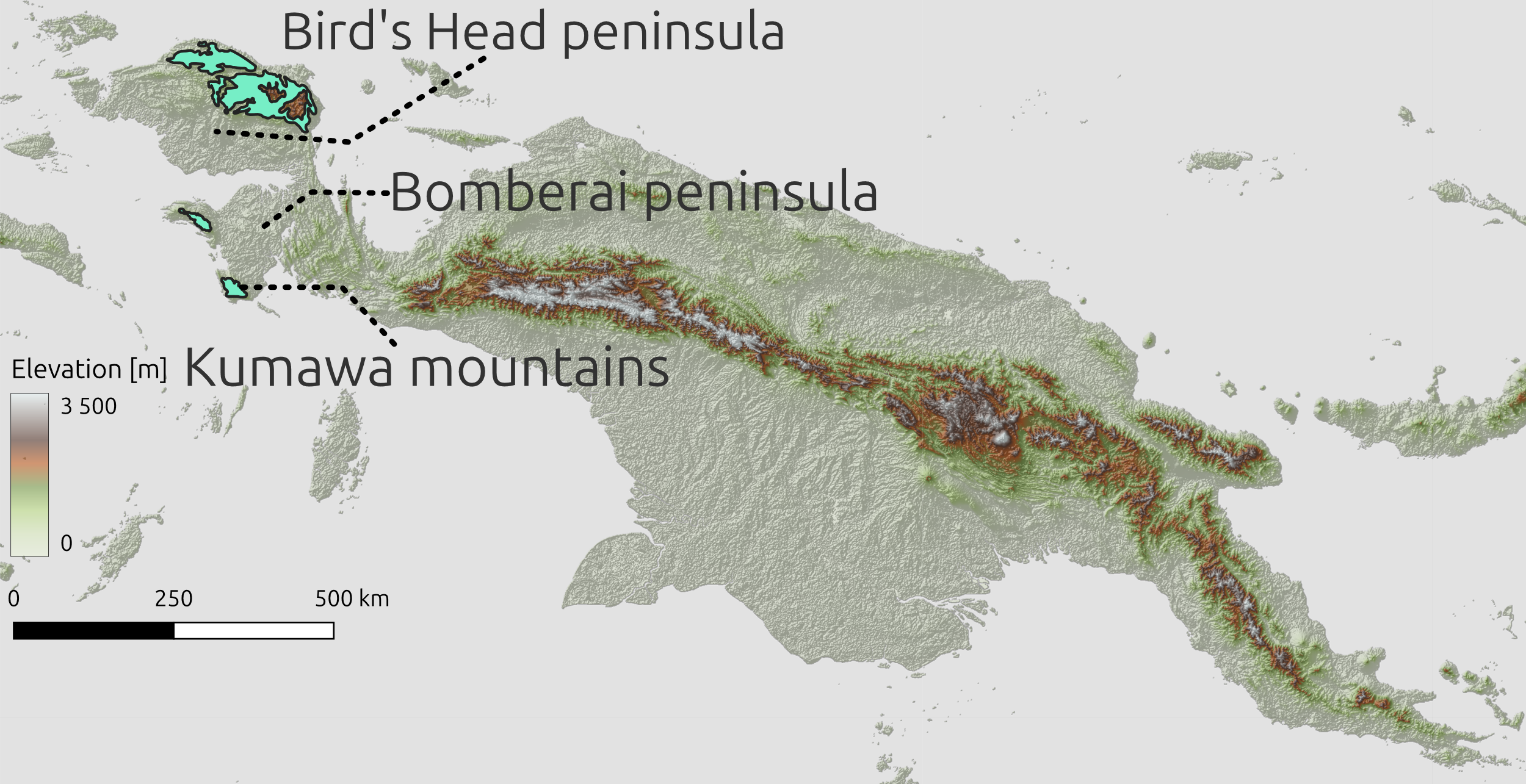


Figure S2. Distribution map of Melidectes leucostephes. Relevant regions from results and discussion are labelled on the map. Distributional data obtained from the IUCN red list (originally hosted by BirdLife). Topographic data was collected from the United States Geological Survey.


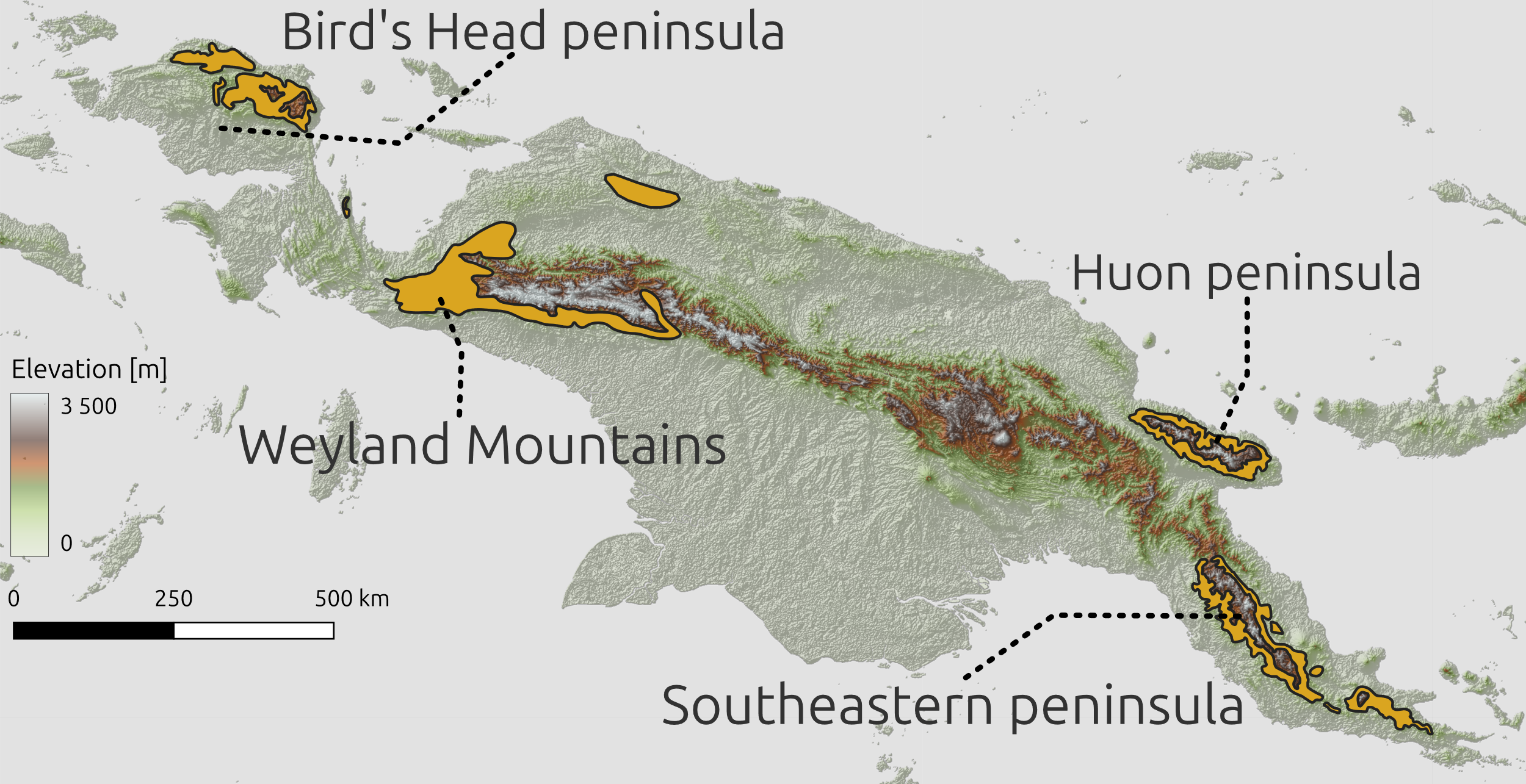


Figure S3. Distribution map of Melidectes ochromelas. Relevant regions from results and discussion are labelled on the map. Distributional data obtained from the IUCN red list (originally hosted by BirdLife). Topographic data was collected from the United States Geological Survey.


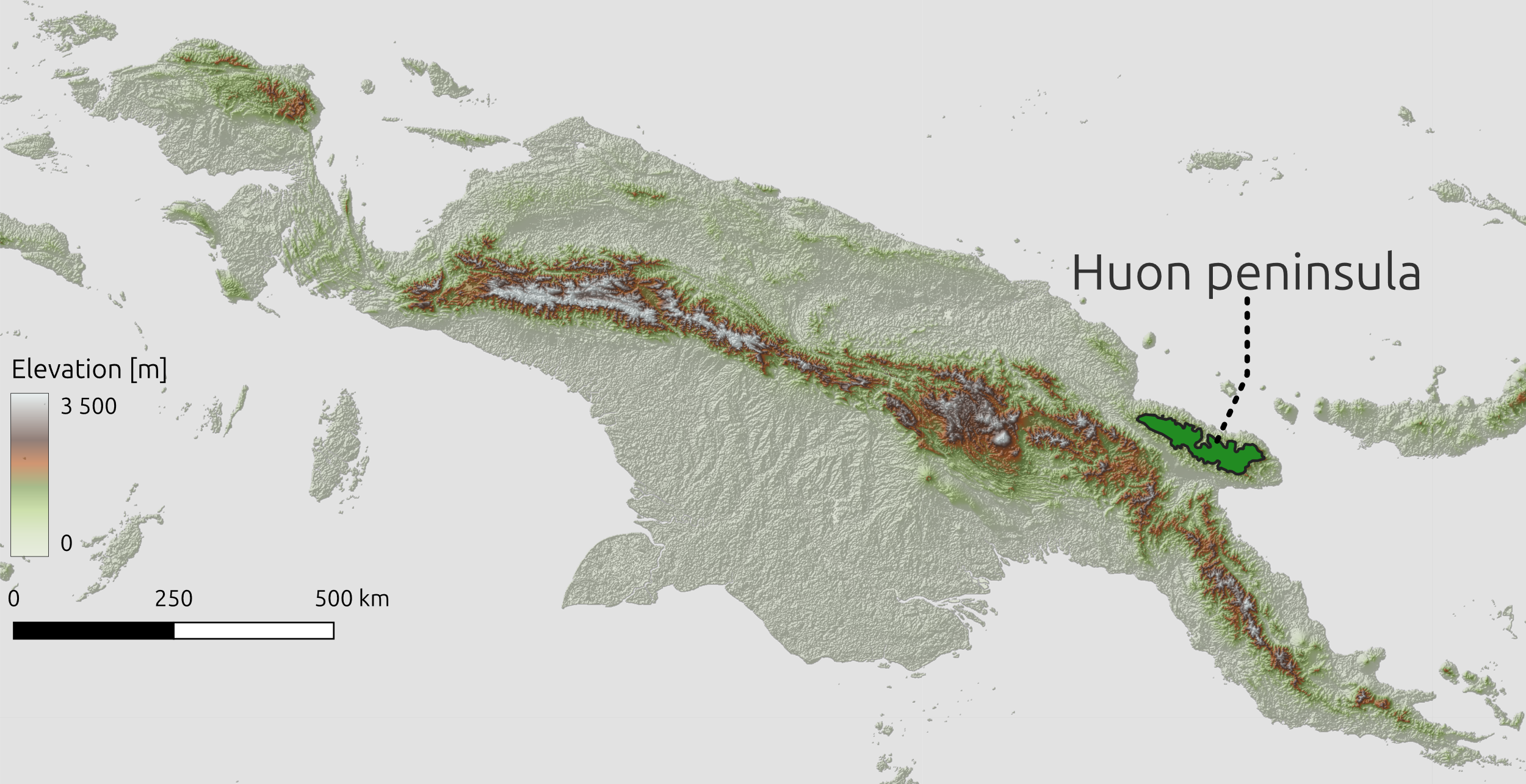


Figure S4. Distribution map of Melidectes foersteri. Relevant regions from results and discussion are labelled on the map. Distributional data obtained from the IUCN red list (originally hosted by BirdLife). Topographic data was collected from the United States Geological Survey.


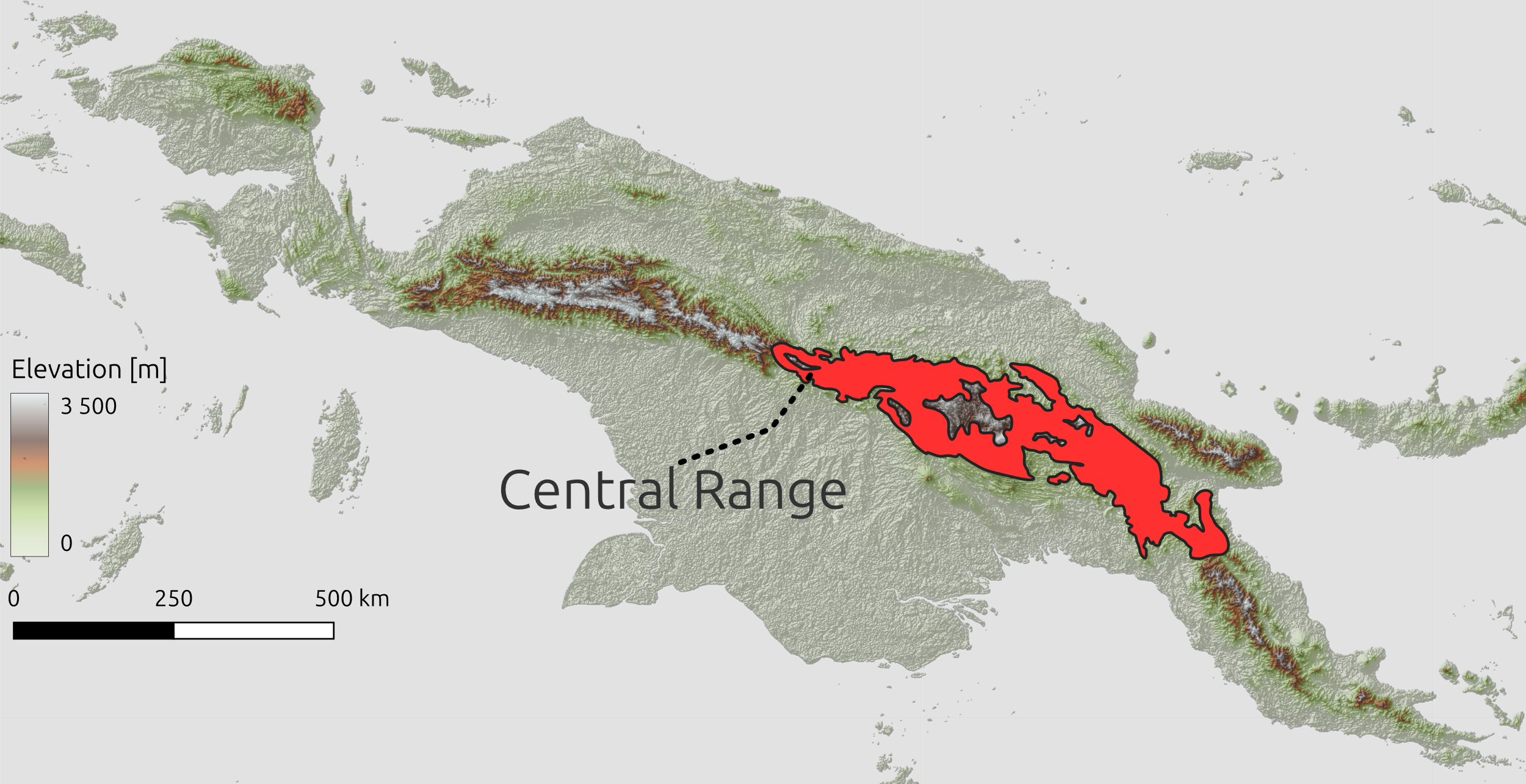


Figure S5. Distribution map of Melidectes rufocrissalis. Relevant regions from results and discussion are labelled on the map. Distributional data obtained from the IUCN red list (originally hosted by BirdLife). Topographic data was collected from the United States Geological Survey.


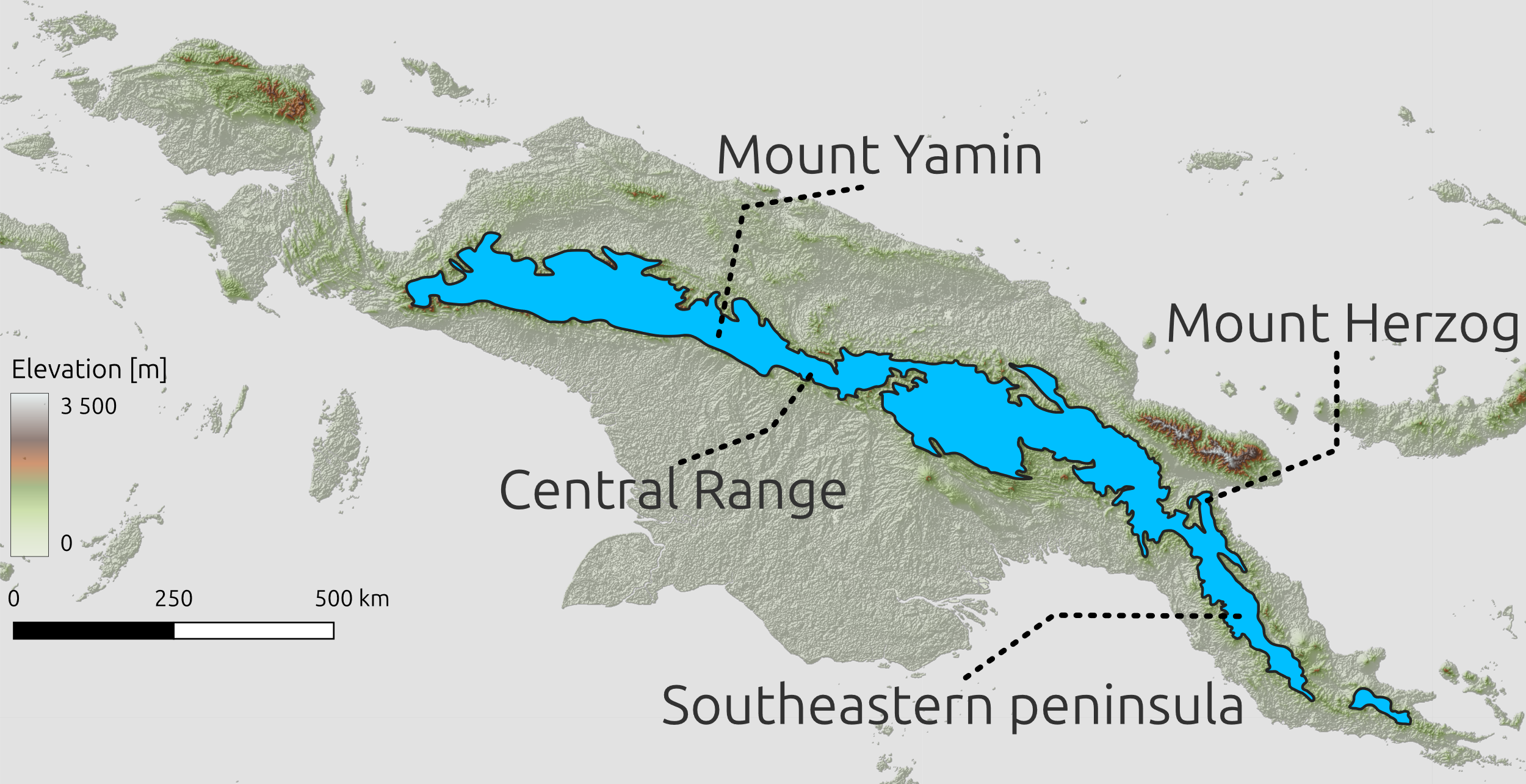


Figure S6. Distribution map of Melidectes belfordi. Relevant regions from results and discussion are labelled on the map. Distributional data obtained from the IUCN red list (originally hosted by BirdLife). Topographic data was collected from the United States Geological Survey.


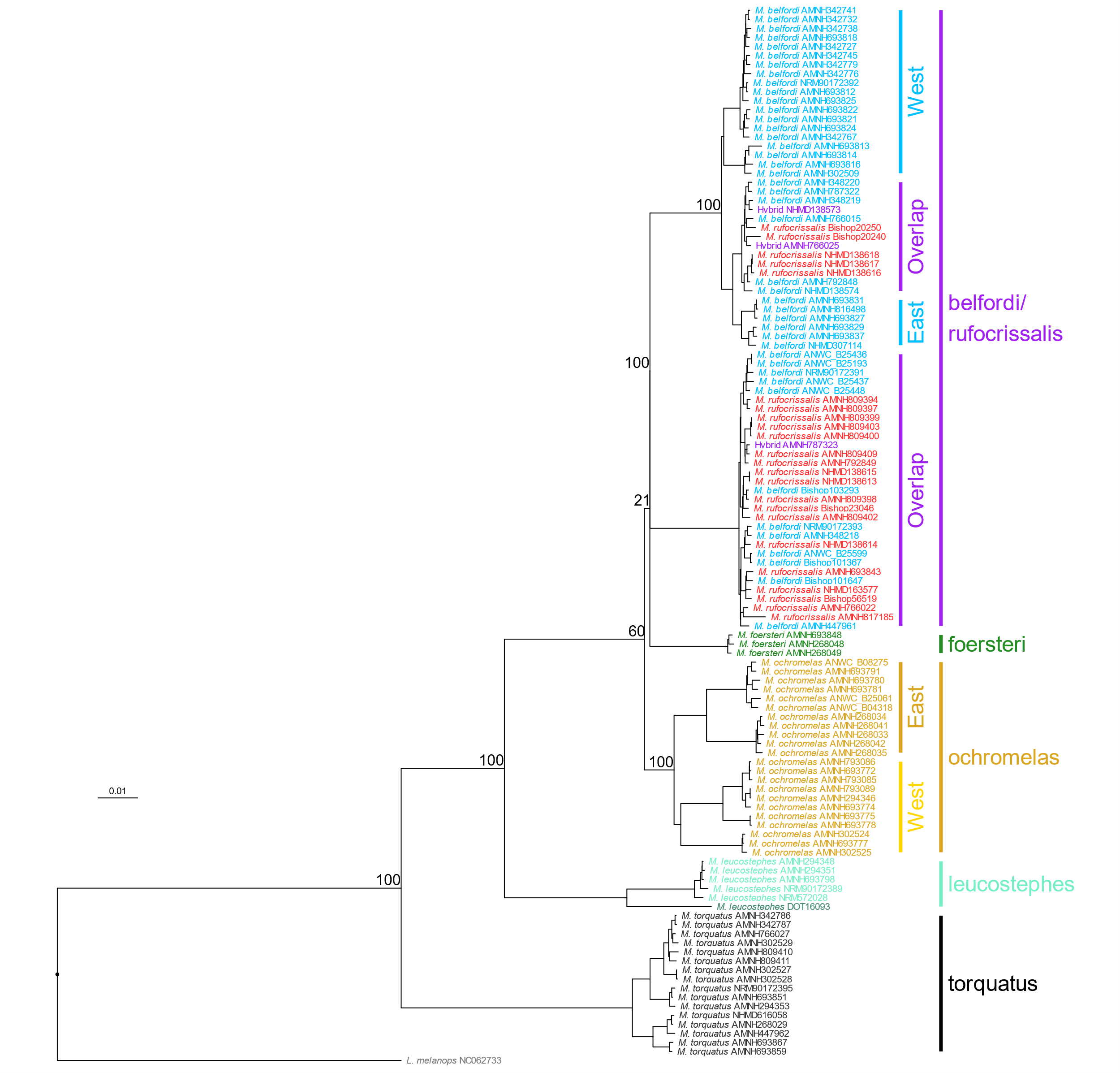


Figure S7. Full mitochondrial phylogenetic tree for all individuals using the Helmeted Honeyeater (Lichenostomus melanops cassidix) as outgroup (labelled L. melanops). Colours and rightmost labels denote different species. Numbers at certain nodes describe bootstrap support values. Additional colours show Hybrids (purple) and M. leucostephes from Kumawa (DOT16093 in darker turquoise). Subdivided labels describe western + eastern populations of M. ochromelas and populations where only M. belfordi occurs (west / east) or where they overlap with M. rufocrissalis.


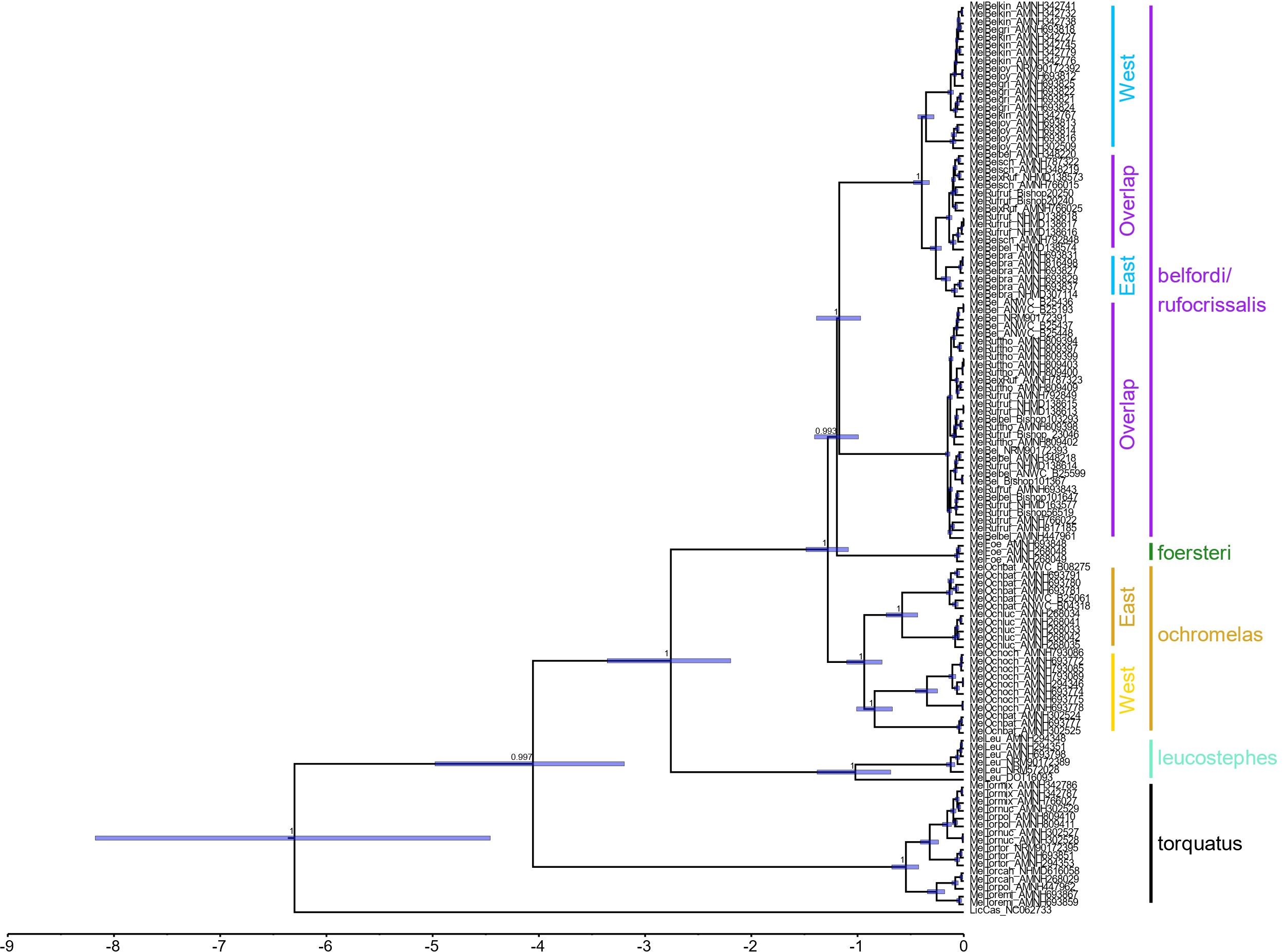


Figure S8. Dated mitochondrial phylogenetic tree based on the BEAST analysis. X-axis shows time in mya. Support values denote posterior probabilities. Error bars represent upper and lower bounds of the 95% highest posterior density interval (HPD) for node heights. Species and subpopulations for important clades are labelled on the right.


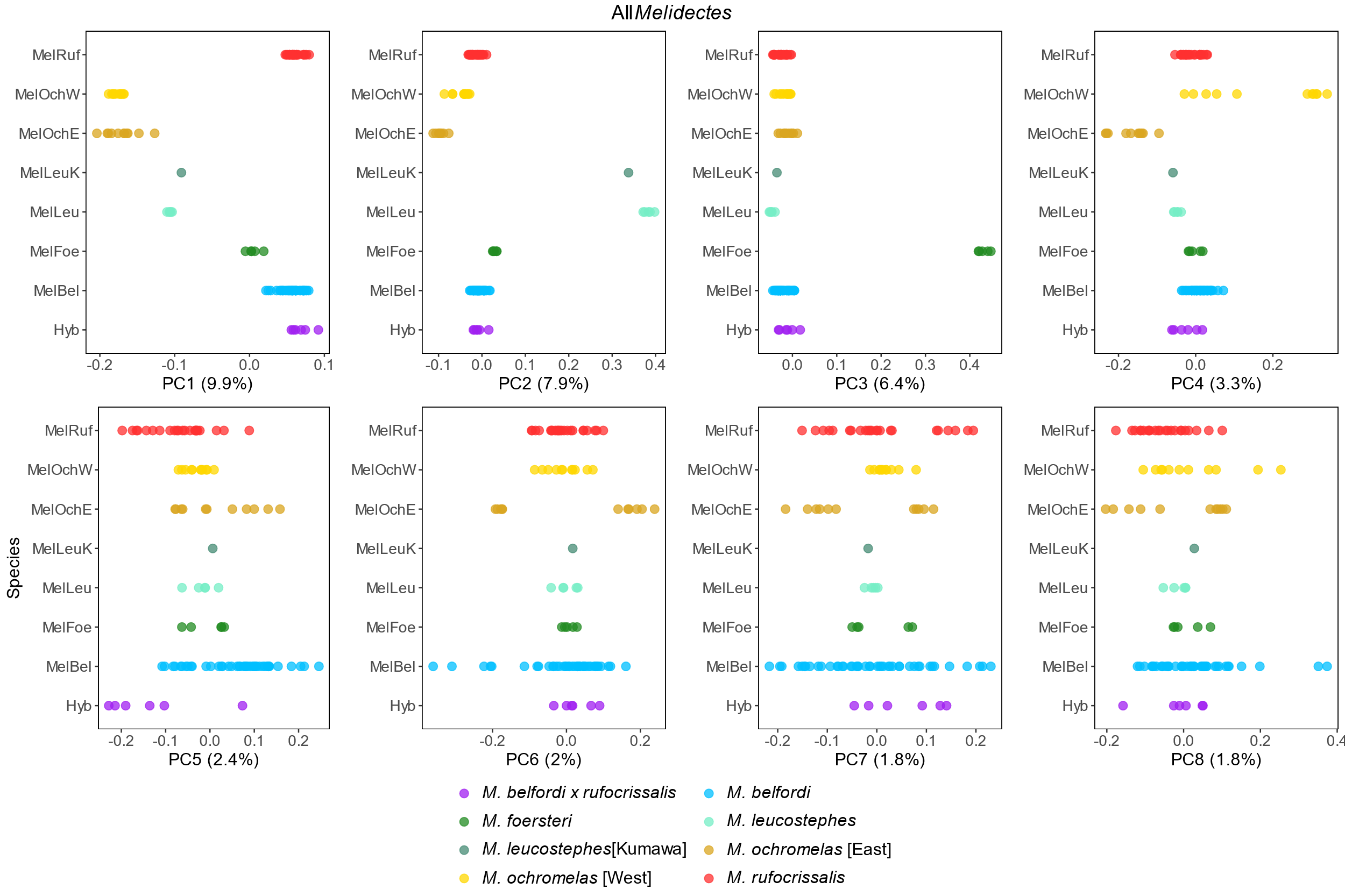


Figure S9. Individual principal components (PC 1 to 8) for all species (minus M. torquatus) and subpopulations of M. ochromelas and M. leucostephes.


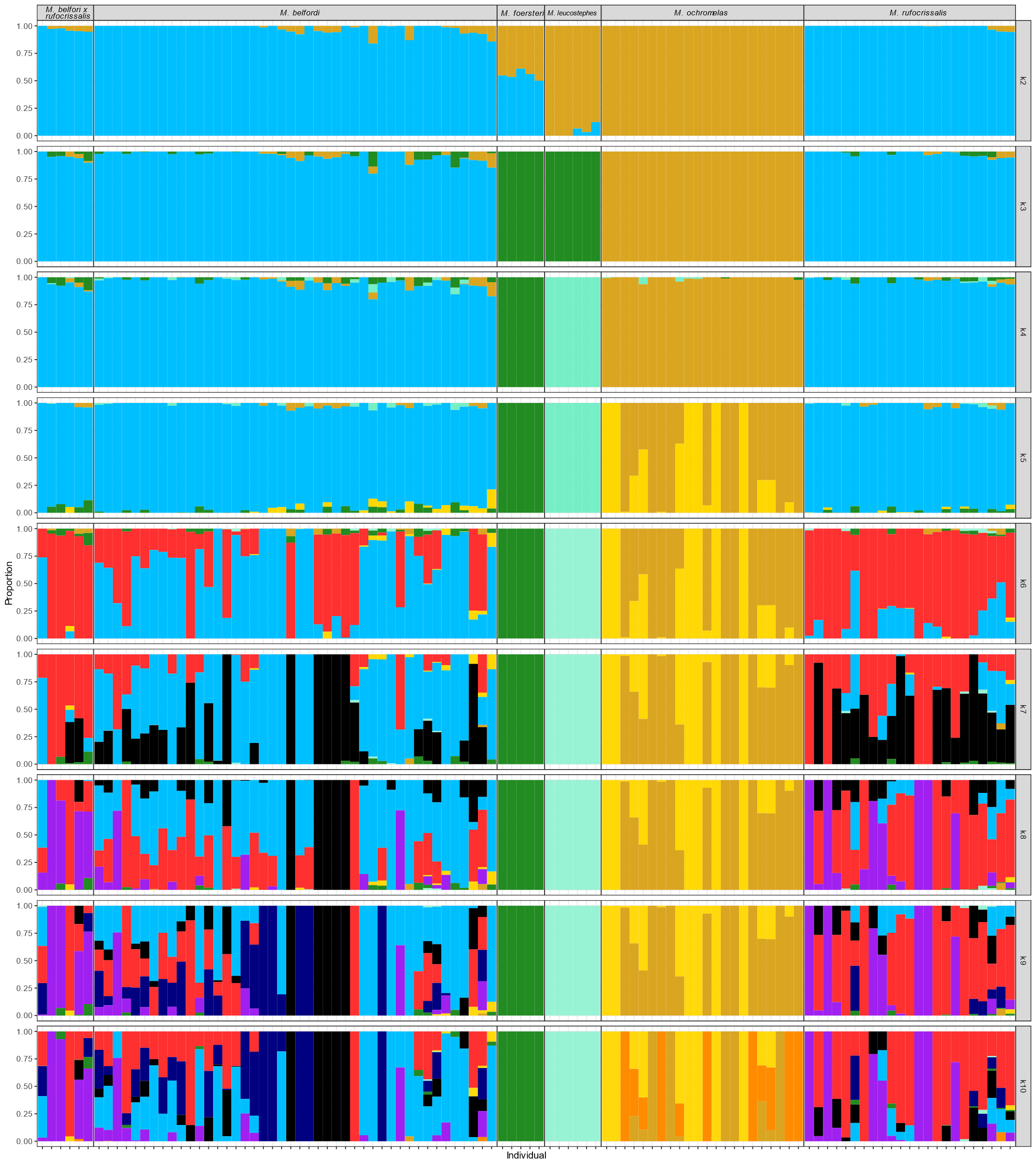


Figure S10. Admixture results from K = 2 to K = 10 for all species (minus M. torquatus). Morphological hybrids of M. belfordi x rufocrissalis are separated in the leftmost group.


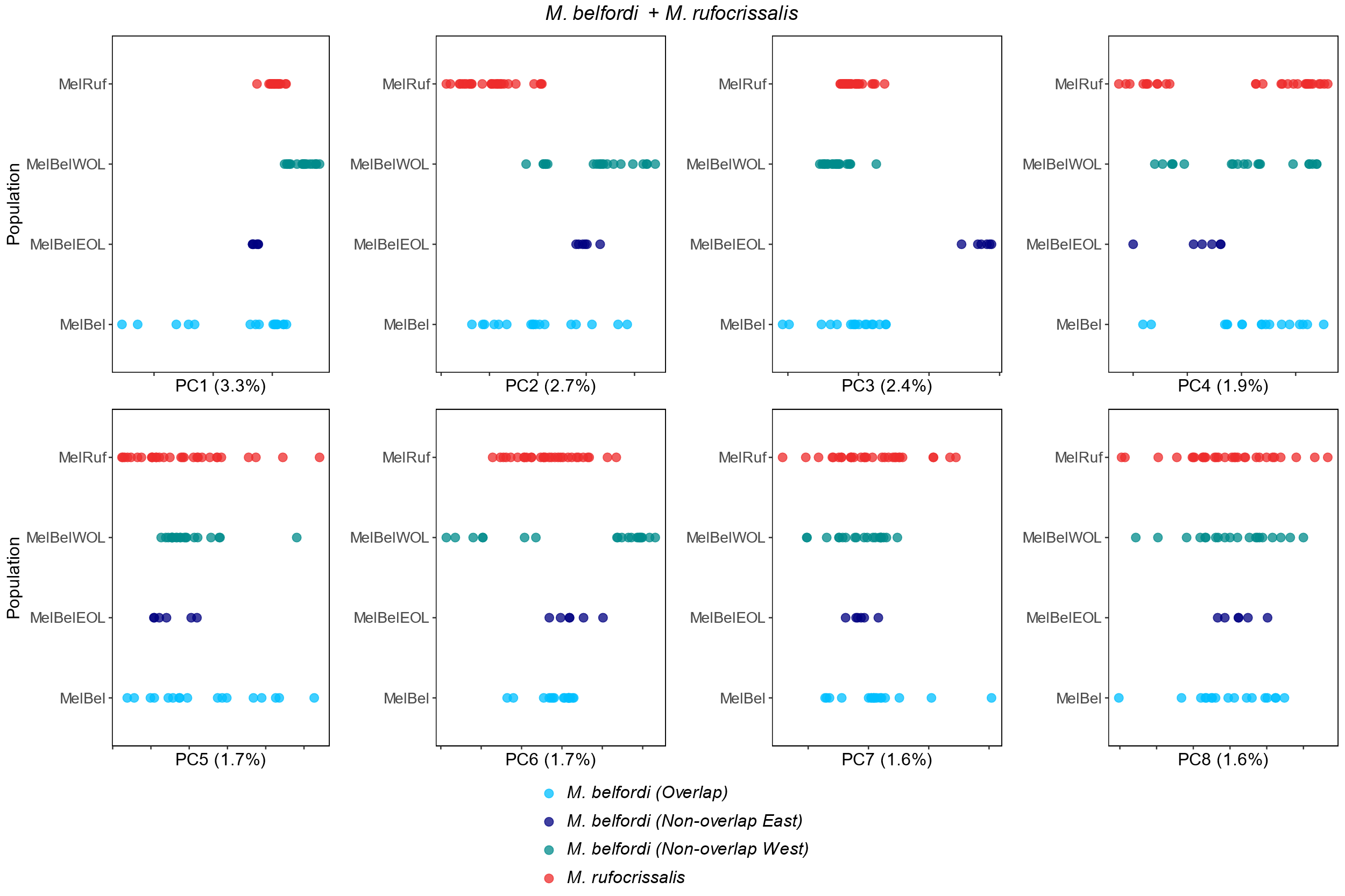


Figure S11. Individual principal components (PC 1 to 8) for M. belfordi + rufocrissalis, separating individuals from western and eastern areas where only M. belfordi occurs (“Non-overlap”) and where M. belfordi overlaps with M. rufocrissalis (“Overlap”).


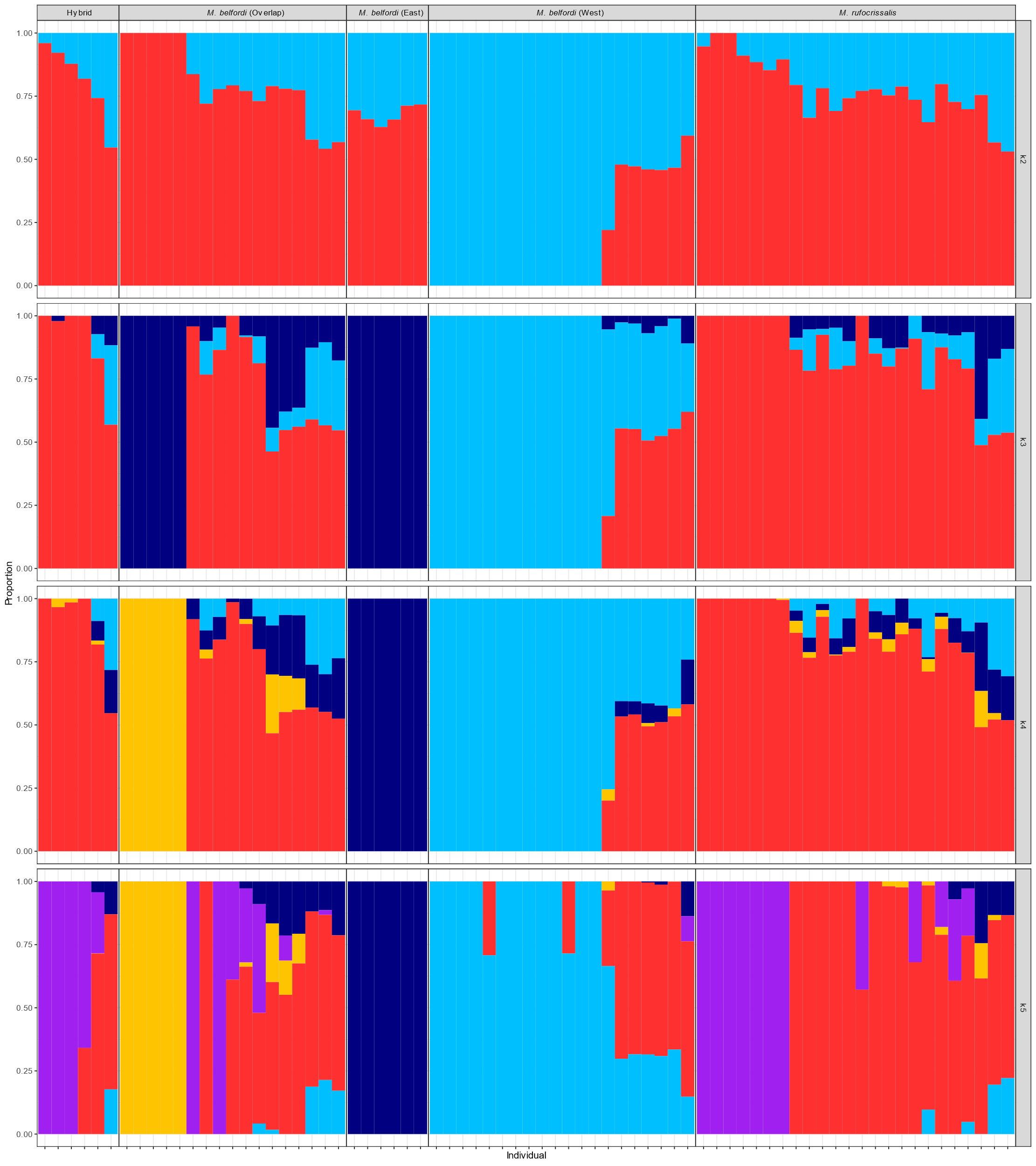


Figure S12. Admixture results from K = 2 to K = 5 for M. rufocrissalis + belfordi. Hybrids as well as western and eastern individuals where only M. belfordi occurs and individuals of M. belfordi that overlap with the distribution of M. rufocrissalis (“Overlap”) are separated.


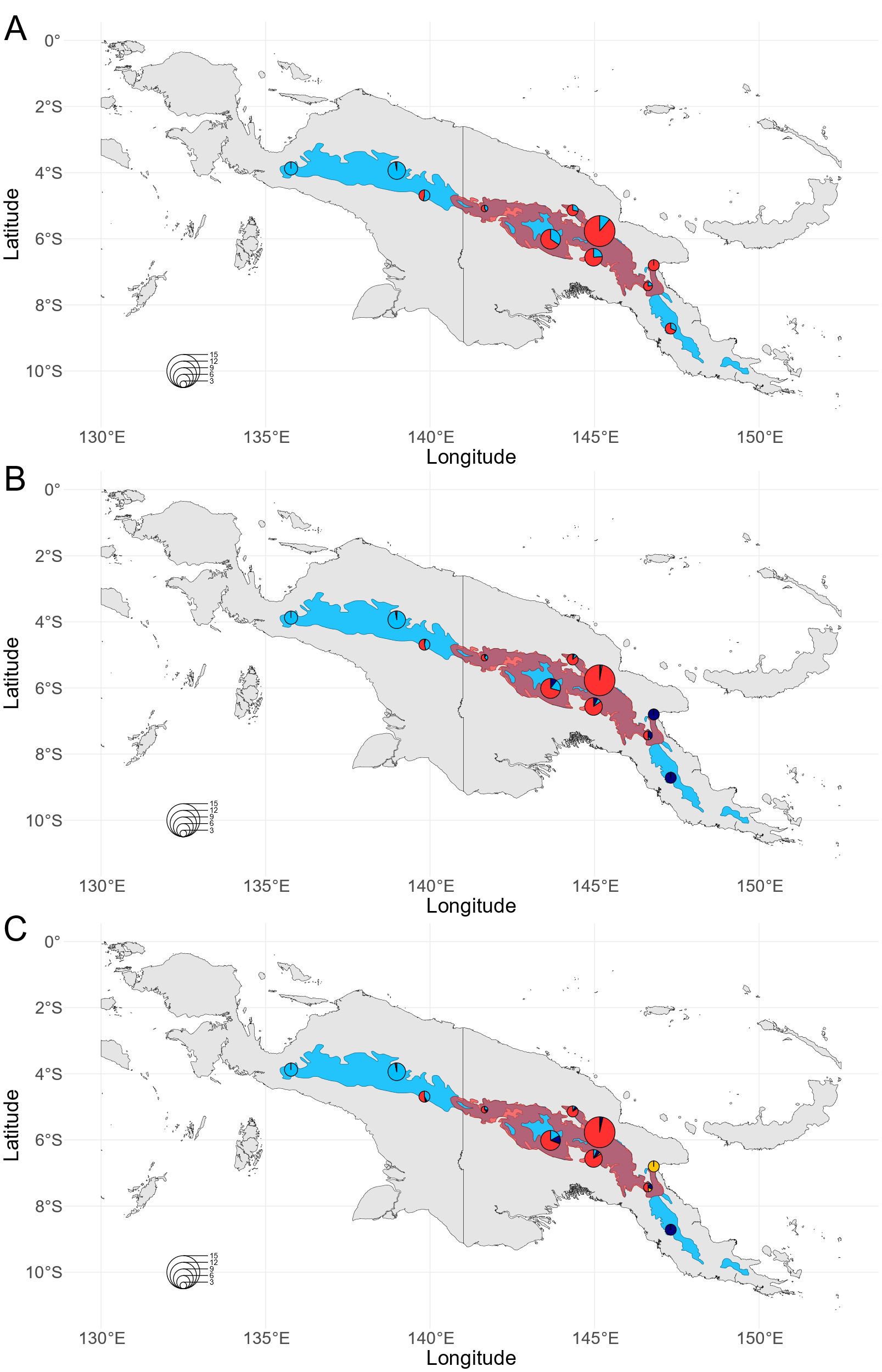


Figure S13. Mean admixture proportions and distributions of M. belfordi (blue) and M. rufocrissalis (red) for K = 2 (A), K = 3 (B) and K = 4 (C). Pie charts are placed for individuals in close proximity and include individuals of both taxa except for areas where only M. belfordi occurs. The size of the pie charts corresponds to the number of individuals.


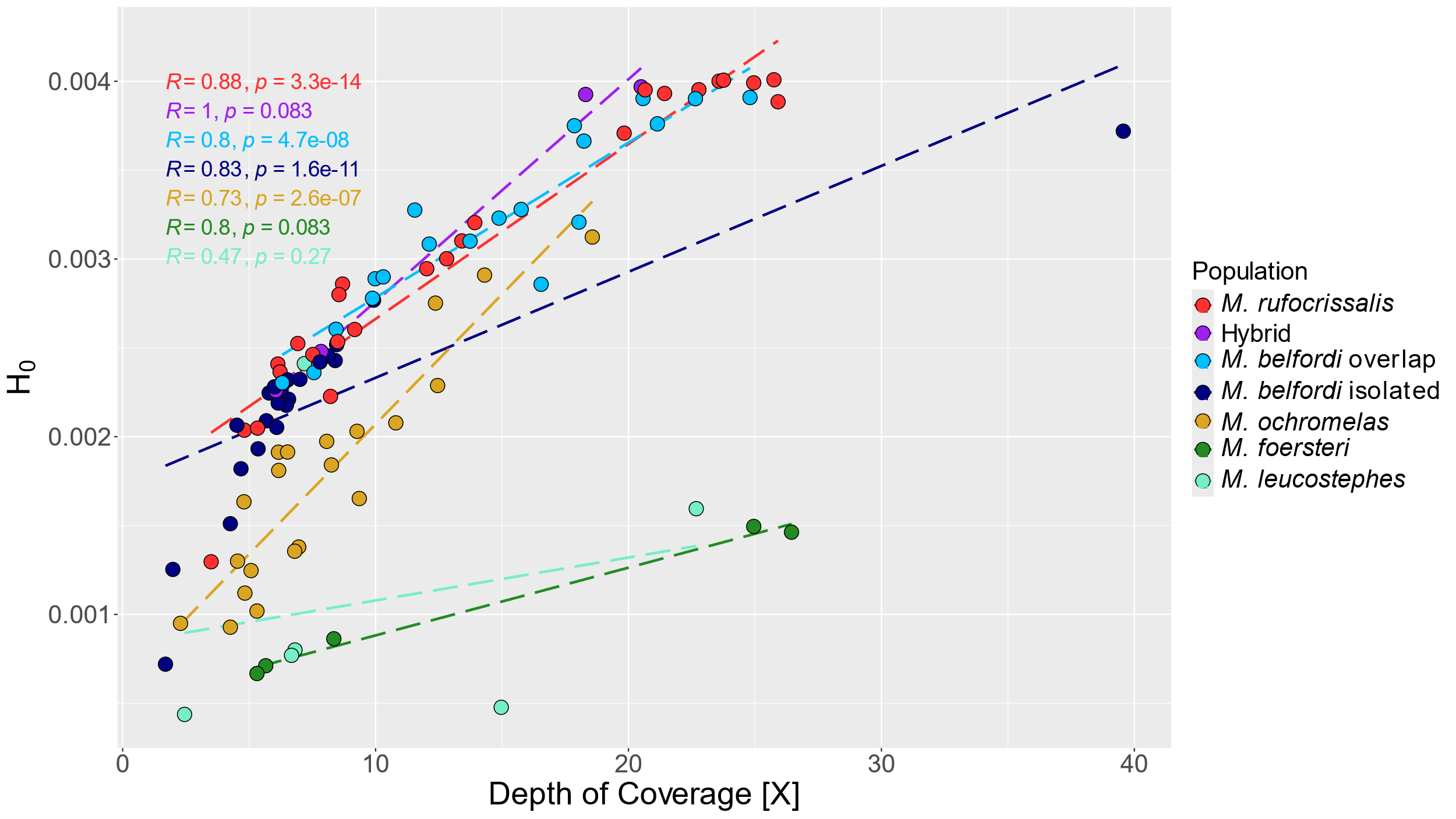


Figure S14. Observed heterozygosity (H_0_) vs depth-of-coverage using linear regression. Kendall’s rank correlation coefficients (R) and p-values were measured through the R package ggpubr and are shown for each subset in the upper left.


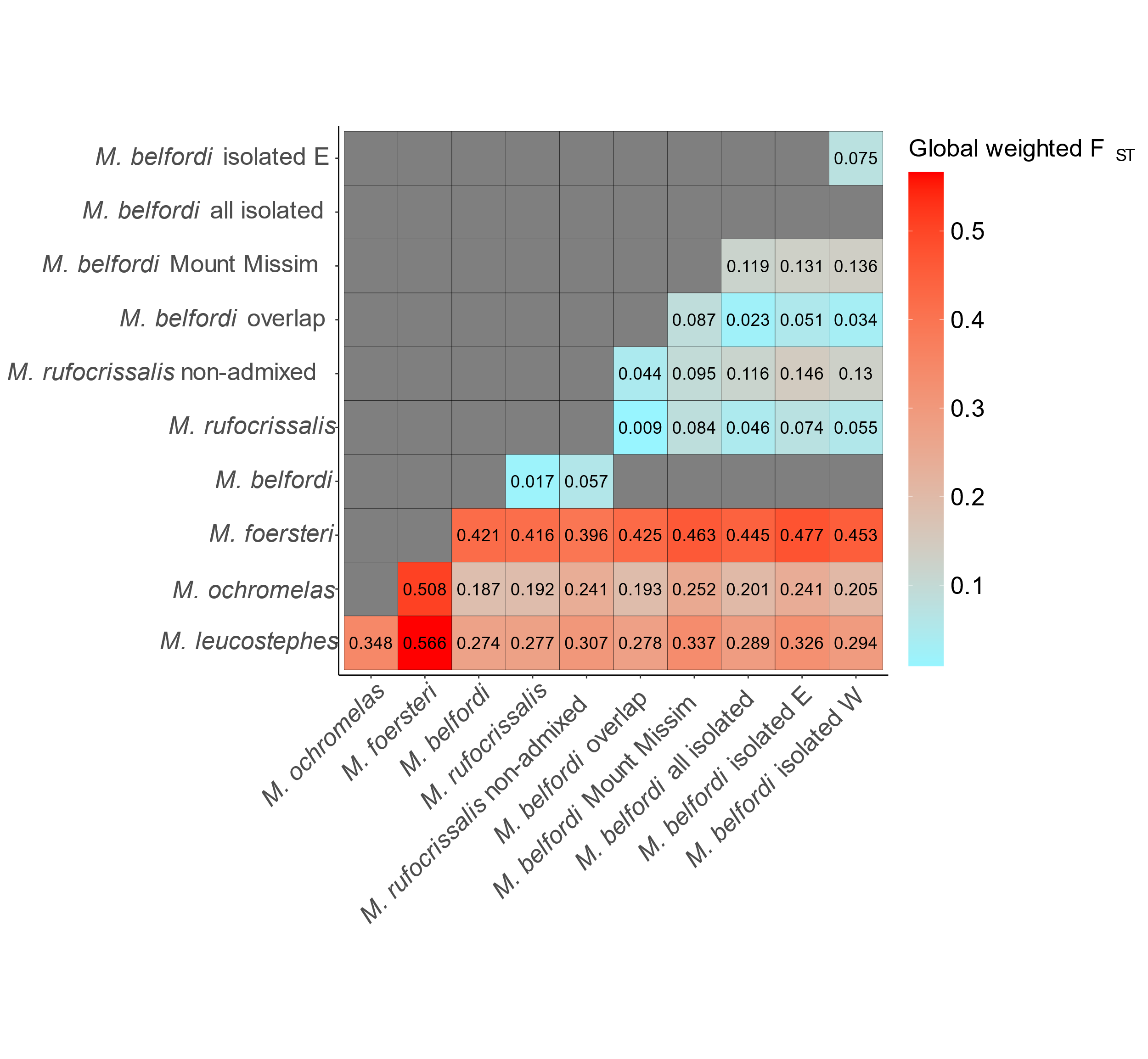


Figure S15. Global weighted F_ST_ for all subsets, greyed out fields are either comparisons of populations with themselves or if a pairwise comparison would be between a population and a subset of the same population. “M. rufocrissalis non-admixed” only included individuals of M. rufocrissalis that showed no admixture at K = 2.


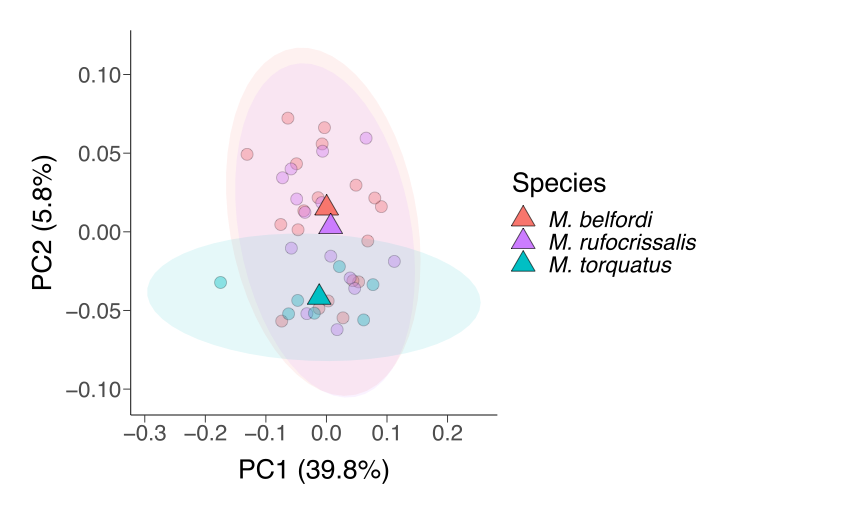


Figure S16. Principal component space (PC1-2) representing overall acoustic similarity between individuals of M. belfordi, M. rufocrissalis and M. torquatus. PC1 and PC2 are averaged syllable scores (constituents of a song) per individual. Each point represents a unique individual. The closer two points are, the more similar the songs of the individuals are to each other. Triangles represent species centroids and ellipses contain 95% of vocalisations of each species.


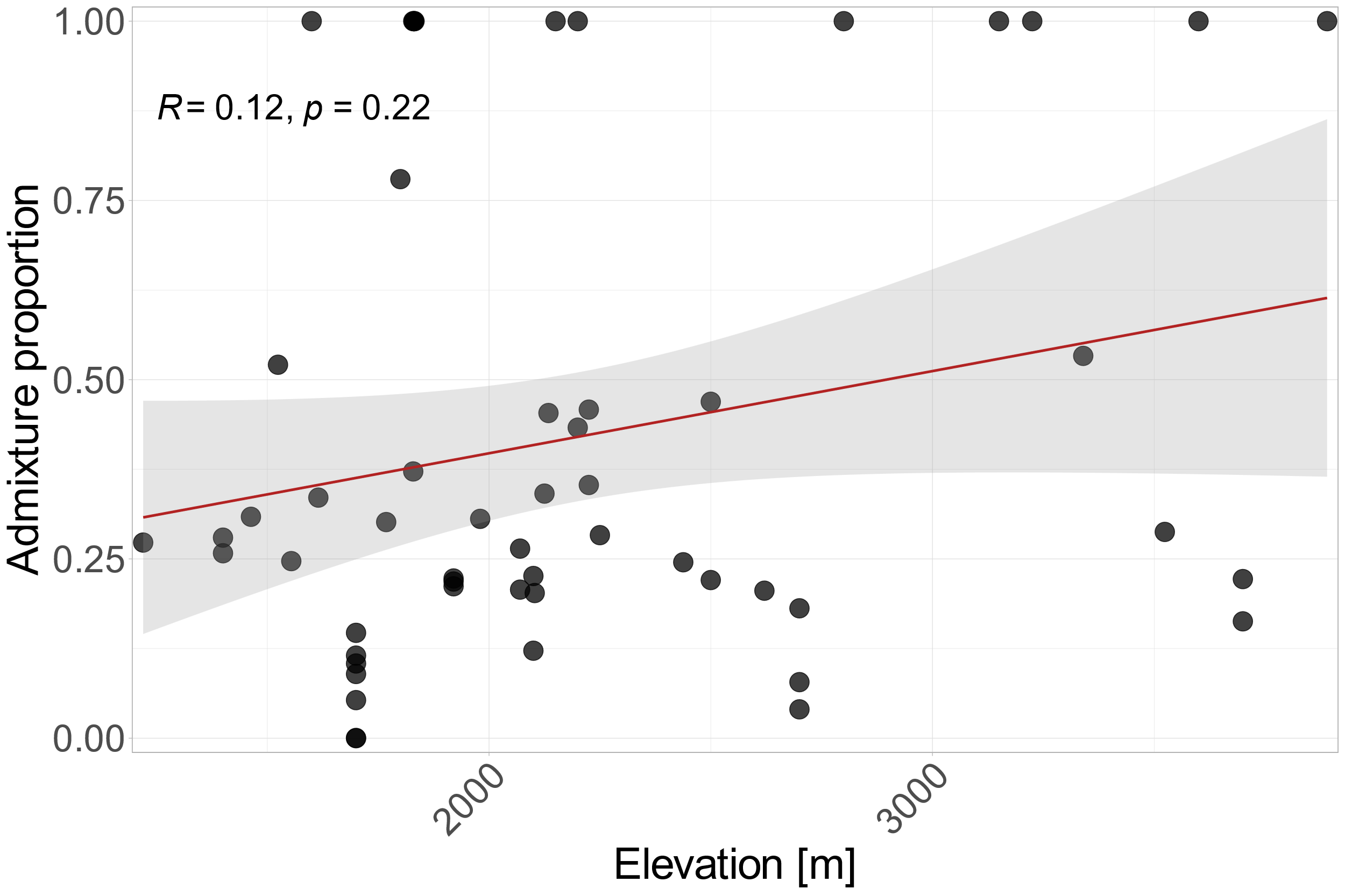


Figure S17. Admixture proportions of M. belfordi and M. rufocrissalis individuals at K = 2 against sampling elevation (Proportion representing the blue cluster at K=2 in S12+S13, i.e. the ancestral cluster which is most prevalent in M. belfordi from western populations [labelled West in Fig. 2A]). Kendall’s rank correlation coefficient and its p-value were measured through the R package ggpubr. Only samples that had elevational information were included.

# Supplementary methods

## Correlating genetic variation to climatic factors (expanded)

*Identifying the climate variable that best explain the observed genetic variation*

We use gradient forest analysis with the R package gradientForest (Ellis et al., 2012) to test which climate variables explain the observed genetic variation best. GF is a machine-learning regression tree-based approach that was originally formulated for detecting species turnover in community ecology datasets and has now been adapted for examining turnover in allele frequency in genomic datasets. For GF modelling, we used 500 regression trees to build a function for each SNP for each climatic variable. Only SNPs with R^2^ > 0 (measure of response of individual SNPs to environmental gradients) were considered predictive loci and were further used in the aggregate turnover functions, accounting for the importance of climatic variables and the goodness of fit for each SNP. For the analysis, we used the 50 k random SNPs drawn from a vcf-file filtered to consist only of variants with a sequencing quality of 30 and observed in at least 90% of all individuals. We also excluded all SNPs with a minimum allele frequency 5% to avoid giving too much importance to rare alleles when looking for loci associated with environmental variation.

The gradient forest analysis provides a ranked list based on the relative predictive power of the environmental variables. To calculate the association between genomic variation and climate variables, we harvested data for the 19 environmental variables for each of the 6,237 2.5 arcmin grid cells from the WorldClim database (Hijmans et al., 2005; http://www.world clim.org/cmip5_2.5m) that fall within the combined distribution ranges of *Melidectes belfordi* and *M.* *rufocrissalis* (BirdLife International and Handbook of the Birds of the World 2022). For each grid cell, the final gradient forest model was used to predict the genomic composition based on the most important, uncorrelated environmental variables indicated in the gradient forest analysis. To visualize the geographic variation in genotype–environment associations, we calculated the principal components of these variables and plotted these using a RGB colour palette based on the first two dimensions as described by Pitcher et al. (2011). Different coordinate positions in the biplot represent differing compositions, as associated with the predictors, and the environmental variables are shown as vectors.

*Predicting phenotypes based on genome–climate associations*

We predicted genome-estimated breeding values (GEBVs) using the R package rrBLUP (Endelman 2011). GEBVs can be interpreted as predictions of ‘latent climate-adapted phenotypes’, that is, unobserved phenotypes assumed to represent local adaptation to particular climate conditions (Gienapp et al. 2020; Lasky et al. 2015). We fitted genome–climate models and predicted climate-associated phenotypes using isothermality (bio3), mean temperature of the driest quarter (bio9) and precipitation of the wettest month (bio13) to construct a multivariable analysis. These climatic variables were shown in the GF analysis to be those that best explain the observed genetic variation among the studied individuals of *Melidectes belfordi* and *M.* *rufocrissalis*. If GEBVs varied along the gradients of the four climatic variables, these associations could reflect genetic responses to differences in the climate conditions across the distribution ranges, that is, local adaptation. Specifically, we used a kinship matrix among individuals combined with their respective climate conditions to calculate the best linear unbiased genomic predictor. In the model, climatic variables were the response variables in a mixed model where genotype random effects had a correlation structure imposed by a kinship matrix among genotypes (Lasky et al. 2015). The kinship matrix was calculated using A.mat functions, and climate-associated phenotypes were predicted with known kinship relative to genotypes using the kin.blup function in rrBLUP.

*Identification of SNPs associated with climate adaptation*

We identified SNPs showing strong associations with the environmental variables identified in the gradient forest analysis based on a latent factor mixed-effect model (LFMM) (Frichot et al. 2013). For LFMM, we ran ten separate Markov chain Monte Carlo runs. We used a latent factor of K = 1 as the admixture analysis indicated that there was no genetic structure among the samples. P values from all five runs were combined and adjusted for multiple tests using a false discovery rate correction of P < 0.05.

*Genetic offset modelling*

The gradient forest analysis was extended to investigate in which part of the geographic distribution the individuals might be most vulnerable to climate change using an extension of the gradient forest analysis as described in Fitzpatrick and Keller (2015). The genomic offset is measured as the mismatch between current and predicted future genomic variation based on genotype–environment associations modelled across the contemporary distribution range. Populations with the greatest mismatch are least likely to adapt quickly enough to track future climate shifts, which potentially can lead to shifts in species ranges, population decline, or even extinction. To measure this, we harvested current and projected future values for each of 19 climatic variables from the 6,237 2.5 arcmin grid cells across the combined distribution range. We downloaded current (1960–1990) and future climate data from the WorldClim v2.1 database (www.worldclim.org). To represent future climate scenarios, we used two CMIP6 one future climate projection (MPI-ESM1-2-HR) with two different emission scenarios (SSP 126 and SSP 585) for 2061-2070. SSP 585 represents the worst-case scenario with an increased mean temperature of 3.5 °C by 2070, while SSP 126 is a more optimistic scenario with an increase of 2.2 °C by 2070. For each grid cell, climatic variables from both current and predicted climates were transformed based on the importance in predicting genomic variation. As a measure of genomic offset, we calculated the Euclidean distance between current and projected future values for each of the 6,237 2.5 arcmin grid cells. Statistical differences in genomic offset between populations were tested with 10,000 permutations to estimate p-values.

RESULTS

The gradient forest analysis identified three environmental variables (of the 19 variables tested and after removing auto-correlated variables with a maximum Pearson's correlation threshold of 0.7, Tables S2 and S3, Figure S18) that explain ca. 60% of the observed genetic variation across the combined distribution of *Melidectes belfordi* and *M.* *rufocrissalis*. These top-three explanatory variables were isothermality (bio3), mean temperature of the driest quarter (bio9) and precipitation of the wettest month (bio13). From the RGB colour plot used to visualize the geographic variation in genotype–environment associations revealed by the gradient forest analysis (Figure S19) it is clear that the precipitation in the wettest month (bio13) is the most important predictor of the genetic response to the environmental conditions in central region of the Central Range (yellowish colours in Figure S19). The purplish colours in the western and eastern parts of the distribution range shows that the variation in the combined effects of the isothermality (bio3) and mean temperature of the driest quarter (bio9) are most responsible for the genetic structure of the individuals living (Figure S19).

To detect local adaptation to a given climate condition, we calculated genome-estimated breeding values (GEBVs) as predictions of ‘latent climate-adapted phenotypes’ using rrBLUP (Endelman 2011; Gienapp et al. 2020; Lasky et al. 2015). We used three climatic variables to reflect the physiological constraints of the species and the trajectory of future climate change (Wallace et al. 2014; Ummenhofer & Meehl 2017), that is, isothermality (bio3), mean temperature of the driest quarter (bio9) and precipitation of the wettest month (bio13). We found the GEBVs to vary along the gradients of these three climatic variables, reflecting adaptation of the climate latent phenotypes to the different climate conditions, that is, local adaptation (Figure S20).

Local climate adaptation can be used to generate correlations between climatic variables and allele frequency because of selection for newly favoured variants or against deleterious variants (Fournier-Level et al. 2011). Using the latent factor mixed model (LFMM) (Frichot et al. 2013) we identified a total of 23 SNPs were found to be significantly associated with bio3, 25 with bio9, and five with bio13.

On the basis of these climate-associated genotypes, we predicted which populations might be most vulnerable to climate change. We calculated local genetic offset as a measure of how much genetic change is needed for a population to adjust to new climate conditions by local adaptation (Fitzpatrick & Keller 2015), that is, without dispersal. We calculated the genetic offset for two emission scenarios for 2061-2070, one worst-case scenario (SSP 585) leading to an increased mean temperature of 3.5 °C by 2070 and one more optimistic scenario (SSP 126) with an increase of 2.2 °C by 2070. We found that under both scenarios the populations in the northwestern parts of the distribution range are the most maladapted to the predicted climate change (Figure S21). Under the more severe scenario (Figure S21 b) the population in the central region of the Central Range will exhibit an extreme environmental stress. The populations in the eastern part of Central range are predicted to be less affected by the climate change under both scenarios.

*Table S2:* Correlations between 19 climatic variables and elevation downloaded for all 6,237 grid cells that fall within the combined distribution ranges of *Melidectes belfordi* and *M. rufocrissalis*. Correlations with an absolute value equal to or higher than 0.7 are marked in yellow.


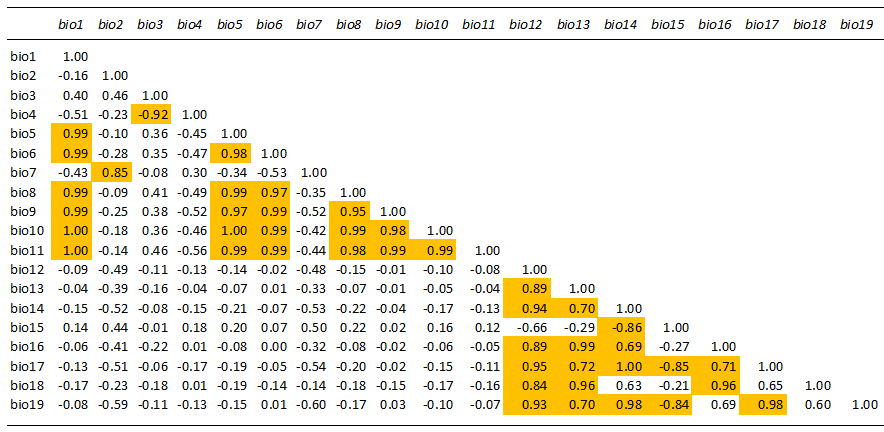


*Table S3:* Definitions of the 19 climatic variables obtained for all 6,237 grid cells that fall within the combined distribution ranges of *Melidectes belfordi* and *M. rufocrissalis*. The variables are listed from most important to least important as indicated by the gradient forest analysis (see Figure 1). Three variables remain after first moving down the list and successively remove all variables that are highly correlated with one or more variables listed above it, and after excluding variables that have only a limited influence on the total genetic variation.


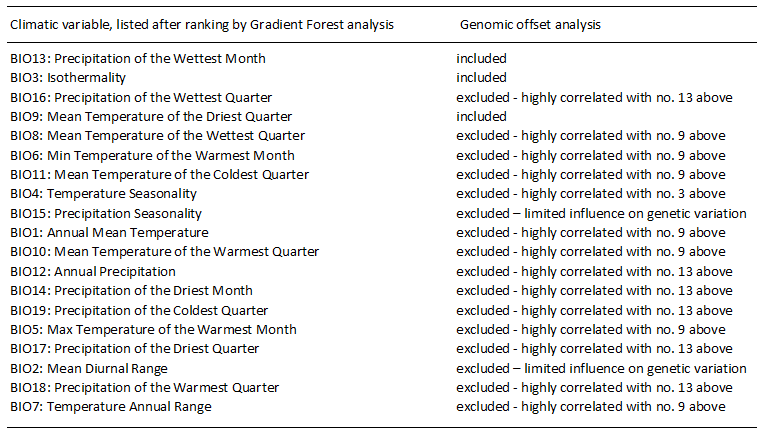


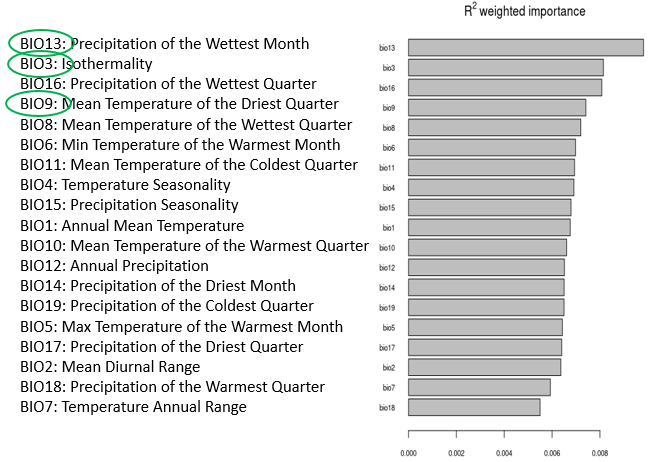


*Figure S18:* The gradient forest analysis ranked the climatic variables in relation to their importance to explain the genomic adaptation across the combined distribution ranges of *Melidectes belfordi* and *M. rufocrissalis*. Three most important variables (marked with green) remain after the removal of less important but highly (p > 0.7) correlated variables, and variables that have limited influence on the total genetic variation (see Tables S2 and S3).


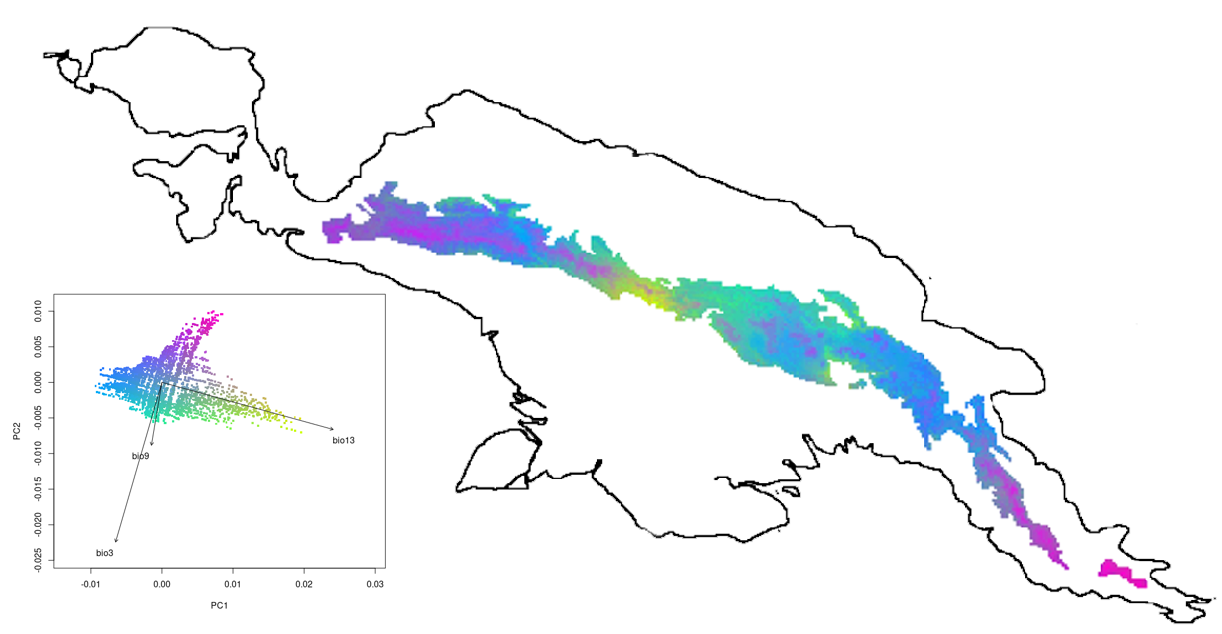


*Figure S19:* Association between genomic variation and climatic variables. The map shows predicted geographic pattern of climate-associated genomic variation along environmental gradients across the combined distribution ranges of *Melidectes belfordi* and *M. rufocrissalis*. The colours are based on the results of the principal components analysis (PCA) of transformed climatic variables. The PCA-based biplot (small panel inset) indicates the contribution of climatic variables to the predicted pattern of genomic variation. The different coordinate positions in the biplot represent differing compositions, as associated with the predictors, and the vector lengths show the loadings of climatic variables on PCA.


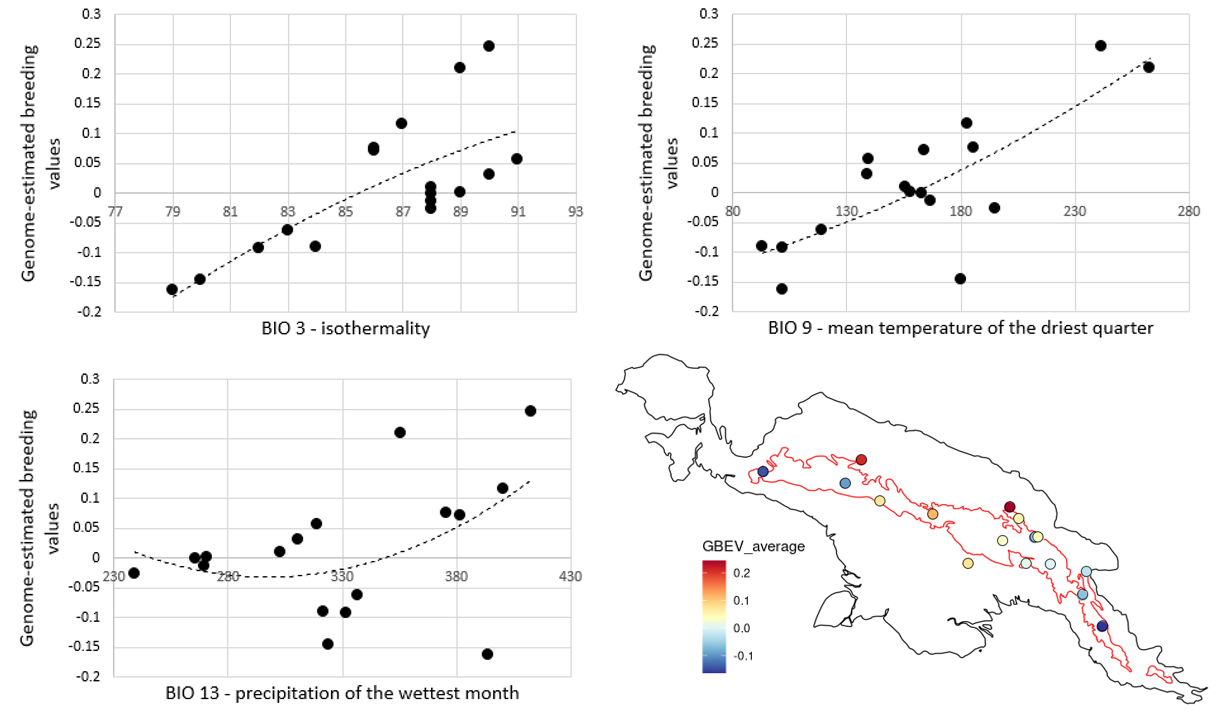


*Figure S20:* The diagrams show that the climatically adapted phenotypes (calculated as genome-estimated breeding values, GEBVs) vary with the gradients of the three climatic variables identified in the gradient forest analysis, which indicates the presence of local genetic adaptation. The map shows the combined distribution range (red outline) and sampling localities for the 62 individuals of *Melidectes belfordi* and *M. rufocrissalis*. The different colours of the localities reflect the predicted genome-estimated breeding values (GEBVs) observed. The uneven geographic distribution of these values is another indication of that the genetic variation cannot be explained by population structure alone.


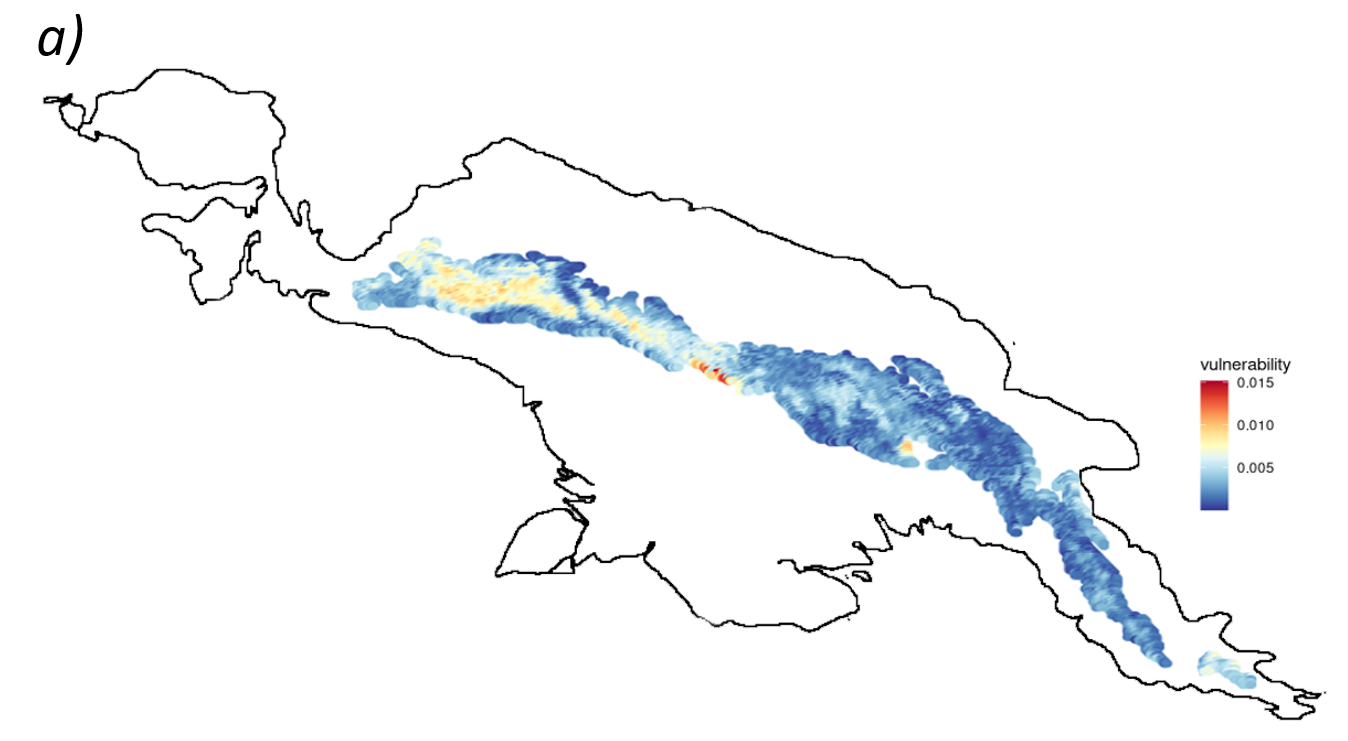


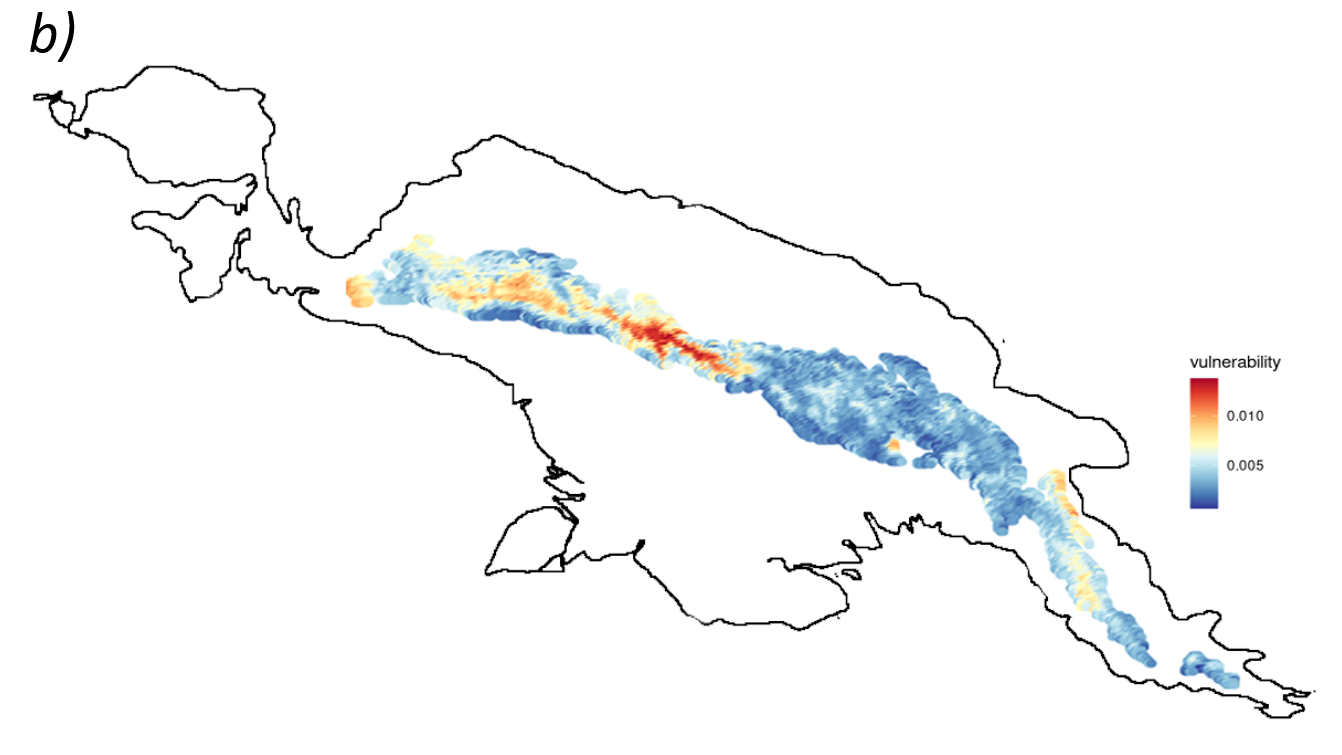


*Figure S21:* Local genetic offset modelling using Gradient Forest analyses of current and predicted future climate conditions. The genetic offset is a measure of how much genetic change is needed for a population to adjust to new climate conditions by local adaptation. Two scenarios are compared, (*a*) 2070 SSP 126 and (*b*) 2070 SSP 585. Both scenarios shows that the populations in the northwestern parts of the distribution range are most vulnerable to the predicted climate change. Under the more severe scenario (*b*) the population in the central region of the Central Range will exhibit an extreme environmental stress.

**References**

- BirdLife International and Handbook of the Birds of the World. *Bird species distribution maps of the world.* Version 2022.2. (2022). Available at <https://datazone.birdlife.org/species/requestdis> .
- Ellis, N. et al. Gradient forests: calculating importance gradients on physical predictors. Ecology 93, 156–168 (2012).
- Endelman, J. B. Ridge regression and other kernels for genomic selection with R package rrBLUP. Plant Genome 4, 250 (2011).
- Fitzpatrick, M. C. & Keller, S. R. Ecological genomics meets community-level modelling of biodiversity: mapping the genomic landscape of current and future environmental adaptation. *Ecol. Let.* 18, 1–16 (2015).
- Fournier-Level, A. et al. A map of local adaptation in *Arabidopsis thaliana*. Science 34, 86–80 (2011).
- Frichot, E. et al. Testing for associations between loci and environmental gradients using latent factor mixed models. Mol. Biol. Evol. 30, 1687–1699 (2013).
- Gienapp, P. et al. Genomic quantitative genetics to study evolution in the wild. Trends Ecol. Evol. 32, 897–908 (2020).
- Hijmans, R. J., Cameron, S. E., Parra, J. L., Jones, P. G., & Jarvis, A. (2005). Very high resolution interpolated climate surfaces for global land areas. International Journal of Climatology, 25, 1965-1978.
- Lasky, J. R. et al. Genome–environment associations in sorghum landraces predict adaptive traits. Sci. Adv. 1, e1400218 (2015).
- Pitcher, C. R., Ellis, N., & Smith, S. J. *Example analysis of biodiversity survey data with R package gradientForest*. (2011). <http://gradientforest.r-forge.r-project.org/biodiversity-survey.pdf>
- Ummenhofer, C. C. & Meehl, G. A. Extreme weather and climate events with ecological relevance: a review. Philos. Trans. R. Soc. B 372, 13 (2017).
- Wallace, J. M. et al. Global warming and winter weather. Science 343, 729-730 (2014).

## Vocal differentiation (expanded)

We sourced 25 recordings of *M. belfordi*, 14 recordings of *M. rufocrissalis*, and 9 recordings of *M. torquatus* from Xeno-canto (<https://xeno-canto.org/>), an online repository of bird vocalizations. The recordings were made in different locations across New Guinea between 1993 to 2019. We focused primarily on recordings labeled as "song," and included some labeled as "uncertain" or "call" if they visually resembled confirmed songs. This approach was used due to the often ambiguous distinction between “songs” and “calls” in species with poorly defined acoustic repertoires. Recordings with identical date and time stamps at the same location were treated as from the same individual, while all other recordings were considered to represent unique individuals. This resulted in a total of 19 individuals for *M. belfordi*, 14 for *M. rufocrissalis* and 7 for *M. torquatus*. The downloaded recordings were converted from .mp3 to .wav files at a 44.1kHz sampling rate for analysis in the acoustic software Luscinia (http://github.com/rflachlan/luscinia, version 2.22.12.01.01). To minimize potential errors from analyzing .mp3 files, we only included high-quality recordings (rated ‘A-C’ on Xeno-canto) (Hay et al., 2024).

All songs were visualised as spectrograms in Luscinia, using a Gaussian windowing function with a window size of 221 samples and maximum frequency of 13kHz. We selected 3 to 47 syllables— the constituents of a song (mean ± SD: 18.22 ± 12.30) per individual. Further, we used the Dynamic Time Warping (DTW) algorithm in Luscinia to estimate the acoustic distance between pairs of syllables. DTW is a commonly used bioinformatic technique in birdsong research that compares two time series sequences by scaling various acoustic features (eg. mean frequency, mean frequency change, etc) to find the optimal alignment between them, resulting in a distance matrix that reflects the (dis)similarity between syllables of a song (Yasukawa et al., 2008; Lachlan et al., 2010; Oñate-Casado et al., 2023). The DTW settings we used were based on established protocols for other songbird species (Rajan et al., 2024; Müller et al., 2024). After running the DTW analysis, Luscinia transformed the output dissimilarity matrix into Euclidean dimensions using non-metric multidimensional scaling and thereafter a Principal Component Analysis (PCA). The PCA allows us to examine the overall patterns of acoustic dissimilarity among syllables of the three species. It is important to note here that the PCA results do not provide specific information on which individual acoustic features contribute to each principal component due to the complex ordination procedures used.

We aggregated PCA scores of syllables per individual and used linear models (‘lm’ function in stats package in R) to assess differences in the principal components among the three focus species in the genus *Melidectes*. We included recording year as a covariate to account for temporal changes in songs due to stochatsic and/or cultural changes over the 26-year recording period. Post hoc tests with Tukey correction were performed using the emmeans package (Lenth 2019). Residuals of the linear models were checked using the Shapiro-Wilk’s test and qq-plots. All statistical analyses were performed in R v4.2.0 (R core team 2022).

**References**

- Hay, E. M., McGee, M. D., White, C. R., & Chown, S. L. (2024). Body size shapes song in honeyeaters. *Proceedings of the Royal Society B*, *291*(2021), 20240339.
- Lachlan, R.F., Verhagen, L., Peters, S., ten Cate, C. 2010, Are there species-universal categories in bird song phonology and syntax? A comparative study of chaffinches (Fringilla coelebs), zebra finches (Taeniopygia guttata) and swamp sparrows (Melospiza georgiana). *Journal of Comparative Psychology, 124:*92-108. <http://dx.doi.org/10.1037/a0016996>
- Lenth R (2022). emmeans: Estimated Marginal Means, aka Least-Squares Means. R package version 1.7.4-1. <https://CRAN.R-project.org/package=emmeans>
- Müller, I. A., Thörn, F., Rajan, S., Ericson, P. G., Dumbacher, J. P., Maiah, G., ... & Irestedt, M. (2024). Species-specific dynamics may cause deviations from general biogeographical predictions–evidence from a population genomics study of a New Guinean endemic passerine bird family (Melampittidae). *Plos one*, *19*(5), e0293715.
- Oñate-Casado, J., Porteš, M., Beran, V., Petrusek, A., & Petrusková, T. (2023). Guess who? Evaluating individual acoustic monitoring for males and females of the Tawny Pipit, a migratory passerine bird with a simple song. *Journal of Ornithology*, *164*(4), 845-858.
- Rajan, S., Lamers, K. P., Both, C., & Wheatcroft, D. (2024). Translocated wild birds are predisposed to learn songs of their ancestral population. *Current Biology*, *34*(11), 2535-2540.
- R Core Team (2022). R: A language and environment for statistical computing. R Foundation for Statistical Computing, Vienna, Austria. <https://www.R-project.org/>.
- Yasukawa, K., Urish, J., Her, A., Light, E. 2008 Similarity in the begging calls of nestling Red-winged Blackbirds. *Journal of Field Ornithology,* *79*(3): 254-262. <http://dx.doi.org/10.1111/j.1557-9263.2008.00171.x>

# Codes and parameters

**Testing substitution models:**

*Modeltest-NG* v0.1.7 (Darriba et al. 2020) was run with the following settings to determine the best substitution model:

modeltest-ng-static -d nt -i $MFA -p 16 -r 23 -t random -T raxml -o $OutFile

**Parameter explanation:**

- -d nt: Set the input data type to nucleotide sequences
- -i $MFA: Specify the alignment file for input
- -p 16: Set number of parallel processes
- -r 23: Use a seed for the random number generator
- -t random: Set the starting topology to a randomly generated tree
- -T raxml: Test only models that are available in RAxML
- -o $OutFile: Specify the output file

**Mitochondrial alignment:**

The alignment was generated using mafft *v7.407* (Katoh and Standley 2013) with the following parameters:

mafft --thread 16 --reorder --adjustdirection –globalpair

--maxiterate 1000 $INPUT

**Parameter explanation:**

-thread 16: Number of threads used

-reorder: Order the output based on alignment similarity

-adjustdirection: Generate reverse complements and align them

–globalpair: Apply Needleman-Wunsch algorithm to generate a global alignment

-maxiterate 1000: Maximum number of iterations. Recommended when

using -globalpair

**Mitochondrial phylogeny through RAxML-NG:**

The actual phylogeny was generated through RAxML-NG v1.1.0 (Kozlov et al. 2019) using these settings:

raxml-ng --all --msa $MSA --seed 1234 --data-type DNA --model GTR+I+G4 --tree pars{10} --bs-trees 100 --threads 16 --prefix $TREE_PRE

**Parameter explanation:**

- --all: Perform both maximum likelihood search and bootstrapping
- --msa $MSA: Specify input alignment file
- --seed 1234: Specify seed for the pseudo random number generator
- --data-type DNA: Set the input data type to DNA
- --model GTR+I+G4: Specify substitution model. We chose a general time reversible (GTR) model accounting for invariant sites (I) and 4 gamma categories (G4)
- --tree pars{10}: Start with 10 parsimony trees
- --bs-trees 100: Run 100 bootstrap replicates
- --threads 16: Specify number of threads for parallelisation
- --prefix $TREE_PRE: Set prefix for output files

**Dated mitochondrial phylogeny with BEAST2:**

We defined the following settings and priors in our input *.xml file to generate a dated mitochondrial phylogeny through *BEAST2* v2.7.4 (Bouckaert et al. 2019):

Site Model:

Gamma Site Model

Gamma category count: 8

Estimate gamma shape

Estimate proportion invariant (starting value 0.6497 as estimated by *modeltest-NG*)

Substitution model: GTR

Estimate all substitution rates except for CT rate (set to 1.0) and estimate nucleotide frequencies

Clock Model:

Relaxed Clock Log Normal

Mean clock rate: 0.0205 (calculated as the average of all rates from mitochondrial regions estimated by Lerner et al. (2011))

Priors:

Tree prior: Coalescent Bayesian Skyline

Markov chained population sizes: Jeffrey’s prior

MRCA prior: All *Melidectes belfordi* and *Melidectes rufocrissalis* individuals were set as monophyletic as we have seen in the maximum likelihood mitochondrial phylogeny and in the autosomal phylogeny.

All other priors were left at default.

MCMC:

Chain length: 100 000 000

Store states every: 10 000

Pre-Burnin: 10 000 000

Number of initialisation attempts: 10

**ANGSD**

**F_ST_ estimation:**

First, sample allele frequency (SAF) files were generated for each population/species with the following parameters/filters:

angsd -bam bamlist.txt -rf $CHRS -out $Out_PRE -doSaf 1 -GL 1 -doGlf 2 -doMajorMinor 1 -ref $REF -anc $REF -doMaf 1 -doCounts 1 -setMinDepth 16 -setMaxDepth 95 -minInd 3 -minQ 20 -minMapQ 20 -uniqueOnly 1 -only_proper_pairs 1 -remove_bads 1 -baq 1 -C 50 -P 10

**Parameter explanation:**

- -bam bamlist.txt: Specify bam as input format, provide a list of all bam files
- -rf $CHRS: Specify which regions to include (scaffolds 1 to 32, representing chromosomes 1 to 29 and chromosome Z)
- -out $Out_PRE: Set prefix for output files
- -doSaf 1: Calculate the Site allele frequency likelihood based on individual genotype likelihoods assuming Hardy-Weinberg-Equilibrium
- -GL 1: Estimate genotype likelihoods using *Samtools*’ model
- -doGlf 2: Generate beagle input file
- -doMajorMinor 1: Infer major and minor from genotype likelihoods
- -ref $REF: Give reference sequence (*M. torquatus*)
- -anc $REF: Give ancestral sequence, as we did not have one available, we used the reference sequence, but following steps need to be performed with -fold 1
- -doMaf 1: Estimate allele frequencies with known major minor
- -doCounts 1: Output the counts of the different bases
- -setMinDepth 16: Discard sites if their total sequencing depth (all individuals added together) is below the given value. I chose 1/3 of the average depth-of-coverage (DoC) times the number of individuals and rounded to a whole number, for example here with 6 individuals and mean DoC of 7.916716:

$$7.916716*6* \frac{1}{3}\approx16$$

- -setMaxDepth 95: Discard sites if their total sequencing depth (all individuals added together) is above the given value. For the upper limit I chose twice the average DoC times the number of individuals and rounder to a whole number, so with the same example as above:

$$7.916716*6*2\approx95$$

- -minInd 3: Only keep sites with at least minIndDepth (default is 1) from at least the given number of individuals, I chose 3 to include at least half of the individuals
- -minQ 20: Minimum allowed base quality score
- -minMapQ 20: Minimum allowed mapping quality score
- -uniqueOnly 1: Remove reads that have multiple best hits.
- -only_proper_pairs 1: Include only proper pairs (pairs of read with both mates mapped correctly)
- -remove_bads 1: Same as *Samtools*’ -x flag which removes reads with a flag above 255 (not primary, failure and duplicate reads)
- -baq 1: Perform base alignment quality (BAQ) computation. Reduces false SNP calling at possibly misaligned bases (Li 2011)
- -C 50: Adjust mapping quality among reads with high numbers of mismatches. For reads mapped with *BWA*, a value of 50 is recommended (according to *Samtools*’ documentation)
- -P 10: Number of threads to be used

Next, 2-dimensional site frequency spectra (SFS) were generated for each relevant species/population pair (using the just generated *.saf.idx files as input) with the following command:

realSFS pop1.saf.idx pop2.saf.idx -fold 1 > ${OutPATH}/pop1-pop2.ml

**Parameter explanation:**

- -fold 1: Generate a folded site frequency spectrum. Necessary when no ancestral sequence is available and the reference is used in its stead

Lastly, F_ST_ estimates for each pair were obtained through

realSFS fst index pop1.saf.idx pop2.saf.idx -sfs pop1-pop2.ml -fold 1 -fstout ${OutPATH}/pop1-pop2 -P 2 -whichFST 1

**Parameter explanation:**

- -fold 1: Generate a folded site frequency spectrum. Necessary when no ancestral sequence is available and the reference is used in its stead
- -fstout: Define prefix of output file
- -P 2: Number of threads to be used
- -whichFST 1: Use Hudson’s estimator to calculate FST as presented in Bhatia et al. (2013)

We ran the following command to obtain global weighted estimates of F_ST_ for each pair:

realSFS fst stats pop1-pop2.fst.idx -fold 1 > pop1-pop2.glob.fst

And we obtained window-based F_ST_ estimates through:

realSFS fst stats2 pop1-pop2.fst.idx -win 100000 -step 20000 -whichFST 1 -fold 1 > ${OutPATH}/pop1-pop2.win.fst

**Parameter explanation:**

- -win 100000: Define sliding window size
- -step 20000: Define step size in which windows are moved

**References**

- Bhatia, Gaurav, Nick Patterson, Sriram Sankararaman, and Alkes L. Price. 2013. ‘Estimating and Interpreting F ST : The Impact of Rare Variants’. Genome Research 23 (9): 1514–21. <https://doi.org/10.1101/gr.154831.113>.
- Bouckaert, Remco, Timothy G. Vaughan, Joëlle Barido-Sottani, Sebastián Duchêne, Mathieu Fourment, Alexandra Gavryushkina, Joseph Heled, et al. 2019. ‘BEAST 2.5: An Advanced Software Platform for Bayesian Evolutionary Analysis’. PLOS Computational Biology 15 (4): e1006650. <https://doi.org/10.1371/journal.pcbi.1006650>.
- Darriba, Diego, David Posada, Alexey M Kozlov, Alexandros Stamatakis, Benoit Morel, and Tomas Flouri. 2020. ‘ModelTest-NG: A New and Scalable Tool for the Selection of DNA and Protein Evolutionary Models’. Molecular Biology and Evolution 37 (1): 291–94. <https://doi.org/10.1093/molbev/msz189>.
- Katoh, Kazutaka, and Daron M Standley. 2013. ‘MAFFT Multiple Sequence Alignment Software Version 7: Improvements in Performance and Usability’. Molecular Biology and Evolution 30 (4): 772–80.
- Kozlov, Alexey M, Diego Darriba, Tomáš Flouri, Benoit Morel, and Alexandros Stamatakis. 2019. ‘RAxML-NG: A Fast, Scalable and User-Friendly Tool for Maximum Likelihood Phylogenetic Inference’. Bioinformatics 35 (21): 4453–55. <https://doi.org/10.1093/bioinformatics/btz305>.
- Lerner, Heather R.L., Matthias Meyer, Helen F. James, Michael Hofreiter, and Robert C. Fleischer. 2011. ‘Multilocus Resolution of Phylogeny and Timescale in the Extant Adaptive Radiation of Hawaiian Honeycreepers’. Current Biology 21 (21): 1838–44. https://doi.org/10.1016/j.cub.2011.09.039.
- Li, Heng. 2011. ‘Improving SNP Discovery by Base Alignment Quality’. Bioinformatics 27 (8): 1157–58. <https://doi.org/10.1093/bioinformatics/btr076>.
