# Supplementary material for: Ephemeral Speciation in a New Guinean Honeyeater Complex (Aves: Melidectes)
Source: Mol Ecol. 2025 Apr 11;34(21):e17760. doi: 10.1111/mec.17760 (PMC12573753; doi:10.1111/mec.17760)
Supplement: Supplementary file 4 — Data S2. FST_plots. [file MEC-34-e17760-s001.zip › README.docx]

Each plot shows a pairwise comparison of two species or subpopulations of *Melidectes. Melidectes belfordi* (Mount Missim) refers to the population of *M. belfordi* by Mount Herzog (*M. belfordi stresemanni*).

Scaffolds are coloured green and black to differentiate neighbouring scaffolds. Numbers on the x-axis correspond to which chromosome each scaffold was strongly associated with through our alignments with *minimap2.* The Z chromosome (present in both sexes in birds, two copies in males) is shown last within each comparison.

FST estimates are based on Hudson’s estimator from Bhatia et al. (2013). The y-axis has been fixed for values between 0 to 1 in all plots. Window sizes are 100 kb in 20 kb steps. The horizontal green line in each plot represents the genome wide / global weighted F_ST_ for each comparison.

**References**

Bhatia, G., Patterson, N., Sankararaman, S., & Price, A. L. (2013). Estimating and interpreting *F* _ST_: The impact of rare variants. *Genome Research*, *23*(9), 1514–1521. https://doi.org/10.1101/gr.154831.113
